# Vietnam Cerebral Palsy Register: protocol for co-design of a national register with people with lived experience of cerebral palsy

**DOI:** 10.1136/bmjopen-2026-116376

**Published:** 2026-07-30

**Authors:** Thi Hong Hanh Khuc, Minh Chau Cao, Tasneem Karim, Thi Lan Anh Dinh, Thi Huong Giang Nguyen, Thi Van Anh Nguyen, Minh Chau Pham, Van Bang Nguyen, Lal Rawal, Sarah Mcintyre, Gulam Khandaker, Elizabeth Jane Elliott, Cerebral Palsy Family Association Vietnam

**Affiliations:** 1 School of Health, Medical and Applied Sciences, Central Queensland University, Rockhampton, Queensland, Australia; 2 Faculty of Medical Technology, Phenikaa School of Medicine and Pharmacy, Phenikaa University, Hanoi, Viet Nam; 3 Cerebral Palsy Alliance Research Institute, Specialty of Child and Adolescent Health, Faculty of Medicine and Health, The University of Sydney, Sydney, New South Wales, Australia; 4 Specialty of Child and Adolescent Health, Faculty of Medicine and Health, The University of Sydney, Sydney, New South Wales, Australia; 5 Cerebral Palsy Family Association Vietnam, Hanoi, Viet Nam; 6 Department of Rehabilitation, National Children’s Hospital, Hanoi, Viet Nam; 7 Medical Faculty, Hoa Binh University, Hanoi, Viet Nam; 8 School of Health Medical and Applied Sciences, Central Queensland University, Sydney, New South Wales, Australia; 9 S.P.O.R.T. Research Cluster, Central Queensland University, Rockhampton, Queensland, Australia; 10 National Centre for Immunisation Research and Surveillance, Westmead, New South Wales, Australia; 11 Central Queensland Public Health Unit, Central Queensland Hospital and Health Service, Rockhampton, Queensland, Australia; 12 Kid’s Research, The Sydney Children’s Hospitals Network, Sydney, New South Wales, Australia

**Keywords:** Protocols & guidelines, Developmental neurology & neurodisability, REGISTRIES, Community child health

## Abstract

**Introduction:**

Cerebral palsy (CP) is the most common physical disability in childhood. Although an estimated 60 000 children live with CP in Vietnam, there is no national register to systematically collect data on the prevalence, risk factors or clinical profile of CP or service access. This paper outlines the protocol for establishing the Vietnam Cerebral Palsy Register (VCPR), a national register which will be co-designed by researchers and clinicians in collaboration with the Cerebral Palsy Family Association of Vietnam (CPFAV). The VCPR aims to inform service planning, policy and research through comprehensive, community-informed data collection.

**Methods and analysis:**

The VCPR will be developed by a consumer-clinician-researcher partnership. Initially, the VCPR will harmonise and pool data from two sources: (1) a hospital-based cohort of 765 children with CP identified at the National Children’s Hospital in Hanoi (NCH) in 2017, and (2) members of the CPFAV, comprising over 4300 children with CP aged <18 years from 63 CPFAV hubs across Vietnam. Subsequently, standardised data for inclusion in the VCPR will prospectively be collected on children newly diagnosed with CP at NCH and other healthcare settings and on children newly referred to CPFAV.

Data will be collected using an adapted version of the Australian CP register questionnaire through a combination of structured caregiver interviews, clinical assessments using validated tools and medical record review. Key demographic variables and information on CP type, motor severity—using the Gross Motor Function Classification System or Manual Ability Classification System, associated impairments (eg, vision, hearing, intellectual disabilities), condition at birth, congenital or other infections, vaccination, educational and nutritional status of the child, access to rehabilitation and family-related factors such as monthly household income, parental education and occupation and maternal health during pregnancy will be collected. The VCPR will contribute data to the Global Low- and Middle-Income Country Cerebral Palsy Register.

**Ethics and dissemination:**

Ethics approval has been obtained from Vietnamese and Australian institutions, whose role is to provide ethical oversight and research governance for the PhD project supporting the VCPR. Written informed consent will be obtained from primary caregivers for data collection, secure storage, access to relevant health records, verification with health professionals and future research contact, with no identifiable data disclosed.

Involvement of people with lived experience of CP is central to the VCPR and leaders and members of the CPFAV will contribute to co-design, governance, participant engagement and dissemination. Findings will be shared regularly with families, clinicians, policymakers and researchers through CPFAV communications, peer-reviewed publications, national and international conferences and the VCPR website to inform equitable service planning, policy and future research.

**STRENGTHS AND LIMITATIONS OF THIS STUDY**

- Strong co-design by people with lived experience-clinicians-researchers and Cerebral Palsy Family Association of Vietnam (CPFAV), ensuring relevance, inclusivity and community trust.
- Large, nationally distributed dataset combining hospital-based and community-based data sources.
- Use of standardised, validated tools aligned with international cerebral palsy registers, enabling global comparability.
- Potential selection bias, with under-representation of children not accessing hospitals or CPFAV networks.

## Introduction

 Cerebral palsy (CP) is described as a group of permanent disorders of development of movement and posture that limit activity and are attributed to a non-progressive injury or abnormality of the developing fetal or infant brain.^1^ The motor disorders characteristic of CP are often accompanied by disturbances of sensation, perception, cognition, communication or behaviour, epilepsy and secondary musculoskeletal problems.^1^ CP is the most common physical disability in children globally^2^ and an estimated 18 million people live with CP worldwide.^3^ Overall birth prevalence is estimated at 1.6 per 1000 live births in high-income countries (HIC).^4^ Based on limited, but increasing available data, the birth prevalence of CP is higher in low- and middle-income countries (LMIC) than HIC.^5–8^

The successive establishment of national CP registers globally has significantly enriched our insights into CP in different geographical regions by providing reliable data about its epidemiology, clinical characteristics, causes and pathogenesis, the interventions used and utilisation of services.^9 10^ Established in the 1950s, the Danish Cerebral Palsy Registry was the first CP register in the world. This research register has documented CP in people born since 1925 and estimated its birth prevalence since 1950.^11 12^ In 2000 and 2008, respectively, the Surveillance of Cerebral Palsy in Europe and the Australian Cerebral Palsy Register (ACPR) were established and these remain active and effective in supporting individuals with CP.^12 13^ These registers facilitate studies of the epidemiology, distribution, frequency and severity of CP, its causes and risk factors and the effectiveness of treatment and prevention strategies and inform planning and evaluation of services.^12 13^

In 2015, the first CP Register in an LMIC was set up in Bangladesh, using available infrastructure from the ACPR.^14^ This was followed by establishment of registers in Nepal, Sri Lanka, Ghana, Indonesia and Latin America and the formation of a network for CP registers in LMICs—the Global Low- and Middle-Income Country Cerebral Palsy Register (GLM CPR, https://www.glmcpr.org/).^15–21^ CP registers can inform service planning and provide a sampling frame for current and future research; however, registers in LMIC often additionally organise or provide services and supports in an approach that differs distinctly from most registers in HIC. Although it is estimated that there are over 60 000 children living with CP in Vietnam,^22–25^ there is no ongoing Vietnam CP Register.

In 2017 we conducted active prospective ascertainment to identify children newly diagnosed with CP presenting to the National Children’s Hospital (NCH) in Hanoi, using an approach modelled on the Paediatric Active Enhanced Disease Surveillance system in Australia.^24^ In total, 765 children newly diagnosed with CP were identified in a 6-month period and data were collected on their demographic characteristics, motor function, clinical severity, associated impairments, immunisation and rehabilitation status and the aetiology of CP if known.^26–30^ We found that children had predominantly spastic CP (95.2%), most were quadriplegic (69.6%) and 60.3% functioned at Level III-V using the Gross Motor Functional Classification System (GMFCS).^26^ Our findings confirmed a higher rate of severe motor functional impairment^26^ than reported in children with CP in HIC settings such as Australia (37%).^13^ Of the children, 76.2% had one or more associated impairments.^26 28^ Full vaccination coverage was lower among children with CP (82.7%) than in children in the general population (96.4%).^30^ Our data showed that a large proportion of the cohort were underweight (28.9%) and stunted (29.0%).^27^ Despite the severity of CP, access to mobility aids and evidence-based treatments was limited.^29^

We estimated that over 40% of the sample had a potentially preventable cause of CP (such as prenatal or postnatal infection, asphyxia, traumatic or accidental brain injury, hyperbilirubinaemia or maternal febrile illness during pregnancy).^26^ This data sets the scene for addressing systemic gaps in prevention, early diagnosis and evidence-based intervention, healthcare infrastructure and public health education. It underscores the urgent need for improved referral, diagnostic and treatment pathways, for clinical practice guidelines and comprehensive support systems that prioritise prevention, timely care and equitable access to services for all children with CP.

Although the NCH-based study provided unique and valuable insights into the clinical characteristics and related factors of Vietnamese children with CP, it captured only children who could access healthcare in a national hospital, potentially excluding children living in institutions, remote or underserved areas. Hospital-based studies also likely over-represent children with severe disease, missing those with milder forms of CP. Many children with CP face barriers to accessing medical care due to financial, geographical or cultural challenges. In our literature review (unpublished), we found that all published studies on children with CP in Vietnam were hospital-based and that no community-based research has been conducted. Establishing a national CP register is crucial to gather comprehensive and unbiased data on prevalence, severity and unmet needs. This data will inform the development of inclusive and equitable healthcare policies and interventions for all children with CP nationwide.

Established in 2017, the Cerebral Palsy Family Association of Vietnam (CPFAV) represents the largest community of people living with CP in Vietnam and their families. The CPFAV is an active member of the Vietnam Federation of People with Disabilities and the International Cerebral Palsy Society. On 21 July 2025, CPFAV had approximately 10 000 members, including 4368 children (aged <18 years) and 347 adults (aged ≥18 years) living with CP and their families. Members are supported by 63 CPFAV ‘hubs’ across 34 provinces in Vietnam, offering in-person support and remote contact, each coordinated by a local hub lead. The CPFAV provides information to, and advocates for, better services for individuals and families with CP. Every CPFAV member with CP is required to provide a medical record that includes confirmation of a diagnosis of CP by a specialist clinician or hospital. With informed consent from families, electronic copies (photographs) of these records are obtained and securely stored at the CPFAV head office.

In partnership with CPFAV, parents/caregivers of all child members will be sent an information letter providing the opportunity to opt out of participation in the Vietnam Cerebral Palsy Register (VCPR). Children whose families do not opt out will be included and assigned a unique VCPR identification number. De-identified summary held by CPFAV will be used to estimate coverage and assess potential selection bias; no identifiable data will be accessed for families who opt out. Prospectively, we conduct clinical assessments on new members in selected but representative hubs, including in community settings and collect data using an updated version of the questionnaire that was used in the hospital-based surveillance study.^24^This collaboration between CPFAV, clinicians and researchers will ensure that the register database includes the core variables used in international CP registers to allow comparison.

This study protocol outlines the development, implementation and evaluation of a CP register in Vietnam modelled on the ACPR and the GLM CPR. Once established, the VCPR database will facilitate research to improve the care of individuals with CP. As with many established CP registers internationally, the VCPR will also serve as a sampling frame for future research, enabling more detailed investigation of specific clinical, social and health service issues among children with CP.^9 21^ The VCPR will also contribute data to the GLM CPR^20 31 32^ which is a multicountry register of children with CP aged <18 years, and share the common goal of GLM CPR: to empower and uplift those living with CP (https://www.glmcpr.org/).

The VCPR is a partnership between Phenikaa University Hanoi, the CPFAV, the NCH and the University of Sydney. Collaborators include the Cerebral Palsy Alliance Australia, Central Queensland University, Hanoi Medical University and the GLM CPR.

## Aims and objectives

Our overarching aim is to develop a national CP register in Vietnam to facilitate research on the prevalence, aetiology, risk factors, access to services, motor type and severity of CP and translate that research into policy and clinical practice. Importantly, register data will also be used to understand the clinical profile of CP in Vietnam to inform treatment and rehabilitation service planning. VCPR will also contribute to the international database of CP registries in LMICs.

Our specific objectives for the register are to:

1. Develop and establish the VCPR to (a) collect a representative, core data set on children with CP from across Vietnam. (b) Establish and populate an enhanced data platform based on the ACPR.
2. Use VCPR data to describe the epidemiology of CP in Vietnam, the demographics, aetiology, risk factors, motor type, functional motor severity according to the GMFCS and Manual Ability Classification System (MACS) and associated impairments, comorbidity and genotype.
3. Assess the availability and use of mobility aids and rehabilitation services for children with CP in Vietnam.
4. Compare clinical characteristics and outcomes of hospital-based and community-based cohorts in the Vietnamese context.
5. Use national data on CP to inform ongoing resource requirements, clinical care, service development, health professional and community education, public health policy and advocacy in Vietnam.
6. To answer specific research questions, the VCPR may also contribute selected de-identified data to the GLM CPR^20 31 32^ and other international research efforts.

## Methods

### Register design and settings

Retrospective and prospective recruitment of a nationally representative cohort of children with CP aged <18 years in a range of community and hospital-based settings in Vietnam including patients of the NCH in Hanoi and members of CPFAV hubs.

### Study participants

#### Case definition

The definition of CP developed by Rosenbaum *et al*^1^ is used: ‘Cerebral palsy (CP) describes a group of permanent disorders of the development of movement and posture, causing activity limitation, that are attributed to nonprogressive disturbances that occurred in the developing fetal or infant brain. The motor disorders of cerebral palsy are often accompanied by disturbances of sensation, perception, cognition, communication, and behaviour, by epilepsy, and by secondary musculoskeletal problems’.

We also accept the following definition which includes five key elements.^1 33^

Cerebral palsy:

1. Is an umbrella term for a group of disorders,
2. Is a condition that is permanent but not unchanging,
3. Involves a disorder of movement and/or posture and of motor function,
4. Is due to a non-progressive interference, lesion or abnormality of the brain, and
5. The interference, lesion or abnormality originates in the immature brain.

#### Inclusion/exclusion criteria

Any child (aged <18 years who meets the case definition for CP as determined by a suitably qualified professional, including a consultant-level physician specialising in general or community paediatrics, paediatric neurology, paediatric rheumatology, paediatric rehabilitation or developmental and community paediatrics), will be eligible for inclusion. For retrospectively identified participants, eligibility for inclusion in the VCPR will be verified using medical records documenting a diagnosis of CP made by a specialist clinician or hospital. The cut-off of 18 years age in accordance with the definition of child used by UNICEF and the United Nations.^34 35^ When appropriate, data will be presented to adhere to the definition of a child as <16 years of age, according to the Law on Children of Vietnam.^36^ In Vietnam, children typically receive care at paediatric hospitals; therefore, hospital-based recruitment will be limited to children aged ≤16 years. However, the use of multiple additional recruitment sources including other healthcare facilities, associations of people with disabilities, CPFAV hubs will facilitate the identification and recruitment of individuals with CP up to 18 years of age, in line with international definitions (eg, UNICEF and WHO), thereby enabling comparability with international data.

#### Participant inclusion

Initially, data from two sources will be pooled to include (1) the cohort of children identified at the National Children’s Hospital in 2017 (all identifying information removed) and (2) all child members of CPFAV who consent to participate in the VCPR/under the opt-out consent process following distribution of an information letter. Subsequently, children newly diagnosed with CP by a medical practitioner in any healthcare facility, associations of people with disabilities and/or joining CPFAV will be enrolled in the VCPR.

1. A national cohort identified in partnership with CPFAV (https://cpfav.org.vn/):

In December 2025, the CPFAV membership included approximately 10 000 individuals comprising 4368 children aged <18 years with CP and their families nationally. All will be invited to contribute their data to the VCPR. The association’s extensive network spans the entire country, from lowlands to mountainous areas and remote islands. CPFAV headquarters is in Hanoi, with branches in Vinh and Ho Chi Minh City and 63 local ‘hubs’ in 34 provinces. The table below presents the number of children with CP who are CPFAV members in each province (table 1). All have medical records that confirm a diagnosis of CP.

**Table 1:** Children with cerebral palsy registered in 63 Cerebral Palsy Family Association of Vietnam hubs in 34 provinces (N=4368; updated 25 December 2025)

No	Province	Number of children with CP	No	Province	Number of children with CP
1	Hà Nội	718	18	Gia Lai	68
2	Ninh Bình	470	19	Sơn La	57
3	TP. Hồ Chí Minh	354	20	Quảng Ninh	56
4	Nghệ An	308	21	Lào Cai	55
5	Hưng Yên	259	22	Quảng Ngãi	39
6	Phú Thọ	226	23	Khánh Hòa	42
7	Hải Phòng	220	24	Lạng Sơn	39
8	Thanh Hóa	222	25	Đồng Tháp	26
9	Đắk Lắk	197	26	An Giang	27
10	Bắc Ninh	195	27	Cần Thơ	25
11	Đà Nẵng	122	28	Cao Bằng	24
12	Thái Nguyên	102	29	Tây Ninh	20
13	Hà Tĩnh	109	30	Huế	19
14	Đồng Nai	89	31	Điện Biên	16
15	Tuyên Quang	80	32	Vĩnh Long	13
16	Lâm Đồng	77	33	Cà Mau	10
17	Quảng Trị	78	34	Lai Châu	6

The number of children registered across CPFAV hubs varies because hubs were established at different times and serve populations of different sizes. The largest hub is located in Hanoi, where the CPFAV head office is based and where CPFAV activities were first established. The figures in table 1 reflect CPFAV membership rather than the prevalence of CP across provinces.

The CPFAV database includes the child’s full name, date and year of birth, sex, address, phone number, Facebook account, economic status, disability certificate, type and distribution of CP, cause of CP if known, GMFCS level, associated impairments and current interventions. Recruitment of participants will commence in 2026.

2. A hospital-based cohort of children with CP previously identified at Vietnam’s National Children’s Hospital by our team:

The database of 765 children with CP^24 26–28^ who comprised the cohort we identified at the NCH in 2017 will also be included in the VCPR.

Data pooling management: Data from the two sources will be pooled, merged, de-duplicated, cleaned, managed, analysed and disseminated by the multidisciplinary study team. Participant data from the community and hospital programmes will be reviewed to exclude duplicate cases by using the child’s national identity number or CPFAV’s identifier. For duplicates, we will retain novel data and resolve inconsistent information.

3*.* Prospectively, data will be collected on children newly diagnosed by a medical practitioner in any healthcare facility and/or children who join the CPFAV.

In the longer-term, data will be collected prospectively through a multicentre recruitment approach involving healthcare facilities, associations of people with disabilities at different levels of the health system across Vietnam, as well as through community-based identification of children who newly join the CPFAV. Healthcare facilities, associations of people with disabilities nationwide will be invited to participate in the VCPR either as data collection hubs or by referring children newly diagnosed with CP and their families for enrolment. This enables capture of children with CP across diverse geographical regions and care settings.

The VCPR may contribute selected de-identified data to the GLM CPR^20 31 32^ and other international research efforts from time to time to address specific research questions. A minimum dataset, which is a subset of the data collected by the VCPR, will be provided to the GLM CPR. These core variables capture key information on CP severity, aetiology, associated impairments and functional outcomes, enabling comparisons across LMICs and pooled data analyses where appropriate.^21 37^ The VCPR may also request data from the GLM CPR to answer research questions. The GLM CPR includes the largest cohort of people with CP in low resource settings globally and is led by one of our study team members (GK).

### Data collection

To support comparison of findings within Vietnam and internationally and/or promote future research collaborations using VCPR data, we will use the original CP Record Form developed in 2017^24^ and used in hospital-based surveillance at NCH. This form includes the core demographic and clinical data fields commonly recorded by established CP registers in Europe, Australia and in LMICs.^5 12–14 20^ For use in Vietnam, the VCPR Record Form was adapted through a co-design process involving Vietnamese clinicians and parents of children with CP (CPFAV members). Additional items included recent hospitalisations, common health conditions (eg, sleep difficulties, constipation) and behavioural concerns. Information on household composition, immunisation status and barriers to vaccination, educational participation and barriers to school attendance and rehabilitation service utilisation and barriers to access is also collected. These variables are included in the GLM CPR and were adapted to align with the local context. These modifications were considered important for understanding healthcare access, participation and unmet needs among children with CP in Vietnam and for informing service planning, advocacy and policy development (VCPR Record Form—online supplemental file 1).

All caregivers of children who are members of CPFAV nationally will be invited to fill in an online, self-administered form (Part A—VCPR Record Form) to provide core demographic information about their children and the family, obstetric and birth history and information on the child’s current educational placement and therapies. Then, they will be contacted by a trained researcher who will collect clinical information and clarify any missing or unclear data. Participants who want to participate in the survey face–face will be invited to attend their local CPFAV hub office. To confirm the clinical characteristics (Part B—VCPR Record Form), parents may be asked to bring their children to a CPFAV’s hub for a clinical assessment and/or to consent to the researcher contacting their health professional and/or accessing their medical records for clinical information that is not available and/or is not known to the parents. This might include information on severity and classification, associated impairments and aetiology and genetic and other test results.

Written informed consent will be collected online from caregivers who provide their information online and on hard-copy forms from those who attend CPFAV hubs in person. The children of CPFAV members who choose not to engage in the project may still receive a clinical assessment if they visit the offices. A hotline will be established to support CPFAV members who have difficulties/concerns while participating in the interview (Tel: +84975220917). Researchers will follow-up on missing data or unclear responses to maximise data completeness. Additional clinical information may be obtained through clinical assessment, consultation with healthcare professionals or review of available medical records with participant consent.

Part A of the VCPR Record Form will be completed by caregivers, with support from trained research assistants where required. Clinical information (Part B) will be collected only by qualified and trained health professionals, including paediatricians, rehabilitation physicians, physiotherapists and general practitioners who have received training in CP assessment and VCPR data collection procedures. CPFAV members with a health-related background may be recruited and trained as research assistants to support participant recruitment and non-clinical data collection activities but will not conduct clinical assessments. Data collected will be reviewed weekly by senior investigators to ensure completeness and accuracy. For children enrolled in the VCPR before the age of 5 years, families will be re-contacted when the child reaches 5 years of age to confirm the CP diagnosis and review previously collected clinical information. This follow-up recognises that the diagnosis, classification, severity and associated impairments in CP may become clearer with age and helps minimise potential misclassification. Children will be identified by their unique national identity number. Children who have not had an identity number (born between 2011 and 2019) will be assigned a unique CPFAV identifier.

### Variables

- Socio-demographic characteristics: Age, sex, parental education and occupation (at birth and current), household income, number of household members (working and dependent members), family history of disability and consanguinity between parents.
- Prenatal and perinatal characteristics: Antenatal care, maternal febrile illness (during pregnancy and labour), maternal nutritional supplementation, pregnancy complications (prolonged labour, malpresentation, pre-eclampsia/eclampsia, postpartum haemorrhage, premature rupture of membranes, preterm labour, maternal fever with or without rash during pregnancy, labour or postpartum), gestational age, birth weight, birth order, multiple birth, birth setting (home/hospital), type of delivery, assistance with conception, early feeding difficulty, signs of birth asphyxia or hypoxic ischaemic encephalopathy, neonatal transfer and neonatal intensive care unit admission, hyperbilirubinaemia requiring treatment and previous live births, stillbirths and/or miscarriages.
- Motor severity and functional activities: Classification using GMFCS level, MACS level, Communication Function Classification System and Eating and Drinking Ability Classification System (EDACS).
- CP description: Type (eg, spastic, dyskinetic, ataxic, hypotonic) and topographical distribution of CP (eg, monoplegia, hemiplegia, diplegia, quadriplegia), timing of origin of CP (pre/perinatal or postnatal), suspected cause (eg, infection, injury, stroke), MRI result and known genetic syndrome or congenital anomalies of the brain.
- Associated impairments: Presence of epilepsy, intellectual disability, visual or hearing impairments, speech difficulties (including identified on Viking Speech Scale) and feeding difficulties (including level of support with EDACS).^28^
- Associated features: growth and nutritional status, including Z-scores and percentiles for current weight for age, height for age, weight for height. For children with severe contractures or scoliosis (GMFCS III-V) where standing height is difficult to obtain, we acknowledge that segmental measures (eg, knee height) or mid-upper arm circumference are preferred alternatives to better reflect actual growth status.^38 39^ Head circumference, growth delay,^27^ gastrointestinal and nutritional problems (gastro-oesophageal reflux disease, constipation, dental caries) and musculoskeletal caries (eg, contractures, scoliosis, hip dislocation) are also recorded.
- Education, rehabilitation and vaccination status: Type of schooling (mainstream/special), reasons for not attending school; access to rehabilitation services and equipment and patterns of rehabilitation use^29^; vaccination status (fully vaccinated or not according to the Expanded Program on Immunisation (EPI) for Vietnam),^40^ receipt of specific vaccines, reason for missed vaccines, presence of BCG scar.^30^ Details of the current EPI for Vietnam,^40^ including vaccine types and recommended vaccination schedules, are provided in online supplemental file 2.
- Other psychosocial and behavioural features: Sleep disorders (difficulty falling asleep, night wakings, sleep quality and duration), behavioural challenges (eg, aggression, self-harm, social withdrawal, repetitive behaviour, emotional dysregulation).

### Training of data collectors

Data collectors will receive training in clinical skills, history taking and assessment, communication skills, maintaining ethical standards and recording and storing data. The first period of data collection will take place over 2 weeks under the supervision of a senior specialist in developmental and child disability. Any concerns regarding clinical findings will be resolved through discussion with senior clinical specialists during data collection.

### Data entry

Multiple quality control measures will be in place for data entry. Each data sheet will be reviewed to identify any missing information, and the family or relevant health professional will be contacted to obtain the necessary data. Once a data sheet is complete, the information will be entered into the VCPR database, with transcription errors corrected during this step. Most fields in the database have predefined response options, allowing unexpected data to be flagged at the point of entry. Additionally, each database record will be independently verified against the original data sheet.

### Data storage, management and security

Information provided by caregivers will be password protected and stored on secure servers hosted by CPFAV (the VCPR online database can be exported to an Excel file). Information collected (via phone or face-to-face) from parents, health professionals and medical records, will be recorded in hard-copy form and securely stored in locked cabinets at the Faculty of Medical Technology, Phenikaa University. It will be transferred to online servers hosted by Phenikaa University and CPFAV. Access to data forms will be restricted to study investigators and authorised personnel. The VCPR database will be backed up daily and will be accessible only to authorised study investigators and designated personnel with password-protected access.

### Data analyses

We will use descriptive statistics and bivariate analyses (χ^2^ and Fisher’s exact test) to describe demographics of all children captured in the VCPR. We will present data on known risk factors, clinical characteristics, associated impairments and access to rehabilitation services in Vietnam, both by region and overall. Descriptive epidemiological measures, including the estimated birth prevalence of CP (per 1000 live births, with a 95% CI), will be derived from the VCPR database. We will compare hospital-based and community-based data to highlight differences in demographics and clinical characteristics. We will also compare our data with international data as appropriate. No individually identifiable information will be included in any reports or future publications. Data analysis will be performed using Stata Statistics software V.16.

**Figure 1:**
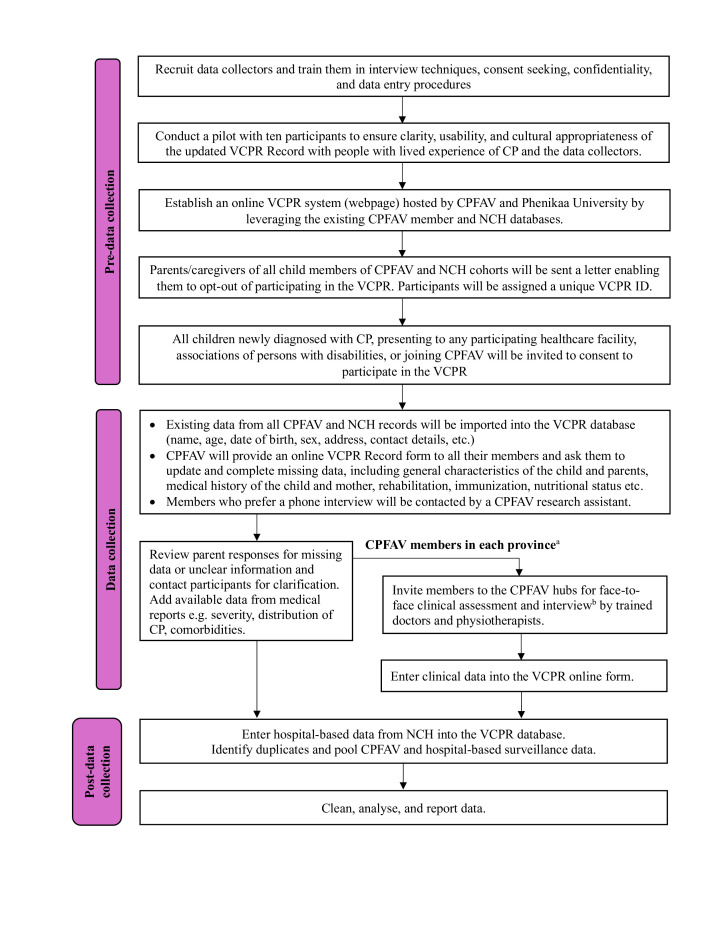
Data collection process. CP, cerebral palsy; CPFAV, Cerebral Palsy Family Association of Vietnam; NCH, National Children’s Hospital; VCPR, Vietnam Cerebral Palsy Register.^a^ The clinical data collection will commence in Hanoi and the process will be followed for other provinces over time with considerations of the local culture and local context. ^b^Members who choose not to participate in the VCPR but want to receive a clinical assessment will still be welcomed.

The overall VCPR data collection process, including participant identification, recruitment, data collection, clinical assessment, and data management procedures is summarised in figure 1.

### Expert advisory and investigative team

The VCPR will be led by a multidisciplinary team of subject matter experts with recognised expertise in CP surveillance, diagnosis, rehabilitation and population-based register development, consistent with international CP register best practice.

Collectively, the investigative team will provide expertise in CP case definition and classification, register governance and management, paediatrics, physiotherapy, rehabilitation, public health and epidemiology, ensuring clinical validity and methodological rigour in accordance with international standards.

### Patient and public involvement of people with lived experience of CP

The VCPR governance framework is founded on a people with lived experience of CP—researcher—clinician partnership. The Inaugural VCPR Advisory Board includes individuals with lived experience of CP in Vietnam working alongside clinicians, researchers and policy-relevant stakeholders. This partnership ensures that the register remains responsive to community priorities, ethically grounded and culturally appropriate.

### Governance and custodianship

The VCPR will be jointly governed by a partnership between the CPFAV, Phenikaa University Hanoi, the NCH Hanoi and the University of Sydney. Collaborators will include the Cerebral Palsy Alliance Australia, Central Queensland University, Hanoi Medical University and the GLM CPR.

The VCPR Advisory Board will act as the independent custodian of the register. The Board will oversee the responsible use, security, maintenance and long-term sustainability of the VCPR database, including oversight of data governance, access, sharing and linkage policies. The Board will provide strategic oversight of register operations, classification and coding practices, regional considerations and future directions and will review all research requests involving identified or de-identified Vietnamese CP data. The Board will also guide the progressive development of the VCPR toward a national population-based CP register.

To ensure objectivity and manage any perceived or actual conflicts of interest, formal governance mechanisms will be implemented. Independent oversight by the Advisory Board will ensure transparency and accountability in decision-making. All register activities will be conducted in accordance with approvals from relevant institutional review boards and applicable national regulations.

Day-to-day management of the VCPR including case ascertainment, data entry, data validation, data cleaning and statistical analysis will be led by Dr Khuc Thi Hong Hanh who will report regularly to the VCPR Advisory Board, relevant government authorities, funding bodies and other key stakeholders, ensuring accountability, data quality and adherence to approved protocols.

### Ethics consideration

Ethics approval for hospital-based surveillance (the existing data of VCPR) was obtained from the University of Sydney Human Research Ethics Committee (HREC) (2016/456) (has extended and is active), Hanoi Medical University (1722/QD-DHYHN) and the NCH (812/QD-BVNTU). Ethics approval for VCPR data collection in partnership with CPFAV the community-based surveillance was obtained from Phenikaa University, Vietnam (No: 024–03.02/DHP-HDDD). Ethics approval was endorsed by Central Queensland University HREC (No: 0000025568).

Informed consent will be obtained by main caregivers (eg, parent). Participation in the VCPR requires consent for the following: (1) the collection, recording, secure storage and reporting of VCPR data, which may involve accessing health records; (2) communication with health professionals to verify or complete VCPR data; and (3) receiving invitations for future research participation. The VCPR Information Form clearly states that no identifiable data will be disclosed. Furthermore, a record will be maintained of families with eligible children who opt out to prevent further contact from register staff.

### Dissemination

This register will be the first to systematically investigate the epidemiology of CP in Vietnam and will provide a large sample. As such, it will be important to share research findings with both Vietnamese and international communities. We will regularly report the results of VCPR data analysis, in CPFAV newsletters/communications, in peer-reviewed journals, by presentation at local and international conferences, to people living with CP, to governments and to other relevant stakeholders. A summary of findings from the pilot phase of participant identification and data analysis will also regularly be made available to all participants through the VCPR website: https://vcpr.org.vn/.

## Limitations and challenges

As with any registry, the VCPR has several limitations and challenges that should be acknowledged. As participation in the VCPR is voluntary, the register may under-represent children with CP who are not connected with CPFAV or participating healthcare facilities and associations of people with disabilities, potentially impacting the completeness required for a population-based register. Establishing and maintaining a national register across multiple provinces will require ongoing coordination, training and resources to support high-quality data collection and long-term sustainability. To improve ascertainment and identify the full range of severity of CP and to support the long-term sustainability of the register, the VCPR will incorporate community-based recruitment pathways alongside healthcare-based recruitment.

## Supplementary material

10.1136/bmjopen-2026-116376 online supplemental file 1

10.1136/bmjopen-2026-116376 online supplemental file 2

## References

[1] Rosenbaum P, Paneth N, Leviton A (2007). A report: the definition and classification of cerebral palsy April 2006.

[2] Oskoui M, Coutinho F, Dykeman J (2013). An update on the prevalence of cerebral palsy: a systematic review and meta-analysis. Dev Med Child Neurol.

[3] Cerebral palsy alliance. https://cparf.org/what-is-cerebral-palsy/facts-about-cerebral-palsy.

[4] McIntyre S, Goldsmith S, Webb A (2022). Global prevalence of cerebral palsy: A systematic analysis. Dev Med Child Neurol.

[5] Khandaker G, Muhit M, Karim T (2019). Epidemiology of cerebral palsy in Bangladesh: a population-based surveillance study. *Dev Med Child Neurol*.

[6] Kakooza-Mwesige A, Andrews C, Peterson S (2017). Prevalence of cerebral palsy in Uganda: a population-based study. The Lancet Glob Health.

[7] Gincota Bufteac E, Andersen GL, Torstein V (2018). Cerebral palsy in Moldova: subtypes, severity and associated impairments. BMC Pediatr.

[8] El-Tallawy HN, Farghaly WM, Shehata GA (2014). Cerebral palsy in Al-Quseir City, Egypt: prevalence, subtypes, and risk factors. Neuropsychiatr Dis Treat.

[9] Hurley DS, Sukal-Moulton T, Msall ME (2011). The cerebral palsy research registry: development and progress toward national collaboration in the United States. J Child Neurol.

[10] Australian Cerebral Palsy Register (2018). Report of the australian cerebral palsy register, birth years 1995–2012. https://cpregister.com/wp-content/uploads/2019/02/Report-of-the-Australian-Cerebral-Palsy-Register-Birth-Years-1995-2012.pdf.

[11] Uldall P, Michelsen SI, Topp M (2001). The Danish Cerebral Palsy Registry. A registry on a specific impairment. Dan Med Bull.

[12] (2000). Surveillance of cerebral palsy in Europe: a collaboration of cerebral palsy surveys and registers. Surveillance of Cerebral Palsy in Europe (SCPE). Dev Med Child Neurol Dec.

[13] Alliance CP Australian cerebral palsy register report 2023.BIRTH YEARS 1995-2016. https://cpregister.com/wp-content/uploads/2023/01/2023-ACPR-Report.pdf.

[14] Khandaker G, Smithers-Sheedy H, Islam J (2015). Bangladesh Cerebral Palsy Register (BCPR): a pilot study to develop a national cerebral palsy (CP) register with surveillance of children for CP. BMC Neurol.

[15] Jahan I, Al Imam MH, Muhit M (2021). Epidemiology of cerebral palsy among children in the remote Gorkha district of Nepal: findings from the Nepal cerebral palsy register. Disabil Rehabil.

[16] Heiyanthuduwage TM, Sumanasena SP, Kitnasamy G (2020). Protocol for the Sri Lankan Cerebral Palsy Register pilot study. BMJ Open.

[17] Jahan I, Bashar SMK, Laryea F (2024). Epidemiology of cerebral palsy among children in Ghana. Afr J Disabil.

[18] Jahan I, Al Imam MH, Karim T (2020). Epidemiology of cerebral palsy in Sumba Island, Indonesia. Dev Med Child Neurol.

[19] Ruiz Brunner M de LM, Jahan I, Cuestas E (2023). Latin American Cerebral Palsy Register (LATAM-CPR): study protocol to develop a collaborative register with surveillance of children with cerebral palsy in Latin American countries. BMJ Open.

[20] Jahan I, Muhit M, Hardianto D (2021). Epidemiology of cerebral palsy in low- and middle-income countries: preliminary findings from an international multi-centre cerebral palsy register. Dev Med Child Neurol.

[21] Goldsmith S, Smithers-Sheedy H, Almasri N (2024). Cerebral palsy registers around the world: A survey. Dev Med Child Neurol.

[22] unicef (2018). Results of the national survey on persons with disabilities in vietnam 2016-2017. https://www.unicef.org/vietnam/media/2776/file/children%20with%20disabilities%20survey%20findings%20vn.pdf.

[23] VUFO-NGO Resource Centre 60,000 vietnamese children suffer from cerebral palsy. http://www.ngocentre.org.vn/content/60000-vietnamese-children-suffer-cerebral-palsy.

[24] Khandaker G, Van Bang N, Dũng TQ (2017). Protocol for hospital based-surveillance of cerebral palsy (CP) in Hanoi using the Paediatric Active Enhanced Disease Surveillance mechanism (PAEDS-Vietnam): a study towards developing hospital-based disease surveillance in Vietnam. *BMJ Open*.

[25] L.TH.H 60,000 vietnamese children with cerebral palsy. https://tuoitre.vn/60000-tre-em-vn-mac-benh-bai-nao-387446.htm.

[26] Karim T, Dossetor R, Huong Giang NT (2022). Data on cerebral palsy in Vietnam will inform clinical practice and policy in low and middle-income countries. Disabil Rehabil.

[27] Karim T, Jahan I, Dossetor R (2019). Nutritional Status of Children with Cerebral Palsy-Findings from Prospective Hospital-Based Surveillance in Vietnam Indicate a Need for Action. Nutrients.

[28] Khuc THH, Karim T, Nguyen VAT (2024). Associated impairments among children with cerebral palsy: findings from a cross-sectional hospital-based study in Vietnam. BMJ Open.

[29] Khuc TTH, Karim T, Cao MC (2025). Rehabilitation status of children with cerebral palsy in Vietnam: findings from a cross-sectional hospital-based study. Disabil Rehabil.

[30] Khuc THH, Karim T, Cao MC (2025). Immunization status of children with cerebral palsy: A cross-sectional hospital-based study in Vietnam. PLoS ONE.

[31] Jahan I, Muhit M, Hardianto D (2021). Epidemiology of Malnutrition among Children with Cerebral Palsy in Low- and Middle-Income Countries: Findings from the Global LMIC CP Register. Nutrients.

[32] Al Imam MH, Jahan I, Muhit M (2021). Predictors of Rehabilitation Service Utilisation among Children with Cerebral Palsy (CP) in Low- and Middle-Income Countries (LMIC): Findings from the Global LMIC CP Register. Brain Sci.

[33] Bax M, Goldstein M, Rosenbaum P (2005). Proposed definition and classification of cerebral palsy, April 2005. Dev Med Child Neurol.

[34] UNICEF Convention on the rights of the child. https://www.unicef.org/child-rights-convention.

[35] United Nations (2022). All persons below the age of 18 years are children: upholding all the rights of all children..

[36] (2016). Law no.102/2016/qh13 dated april 05, 2016 “law on children”.

[37] Katangwe TJ, Kruger M, Chimowa T (2024). Variables included in cerebral palsy registries globally: A scoping review. Dev Med Child Neurol.

[38] Jahan I, Ruiz Brunner M de LM, Muhit M (2023). Novel weight estimation equation for children with cerebral palsy in low-resource settings: Validation in a population-based cohort. Dev Med Child Neurol.

[39] Atar A, Pulat Demir H, Şahin Anılgan İN (2026). Beyond BMI: functional-level based nutritional and body composition assessment in children with cerebral palsy. Disabil Rehabil.

[40] (2017). Circular 38/2017/tt-byt introducing lists of infectious diseases, scope and recipients of compulsory vaccines and biologicals [in vietnamese].

