# Supplementary material for: Vietnam Cerebral Palsy Register: protocol for co-design of a national register with people with lived experience of cerebral palsy
Source: BMJ Open. 2026 Jul 30;16(7):e116376. doi: 10.1136/bmjopen-2026-116376 (PMC13423169; doi:10.1136/bmjopen-2026-116376)
Supplement: online supplemental file 1 [file bmjopen-16-7-s001.docx]

**Annex 1. Vietnam Cerebral Palsy Register form (PART A)**

**General information and medical history factors**

**General information**

Full name:.………………………………………………………………………………………….

Gender:  Male  Female DOB (DD/MM/YYYY): _ _ /_ _ / _ _ _ _

Place of birth:………………………………………………………………………………………

Ethic group:  Vietnamese  Other, please specify: …………………………………………….

Address:…………………………………………………………………………………………….

House no.:.…………….. Street name:.…………………………District:.……………………….

Province:.………………………………………………………………………………………......

Caregiver:  Mother  Father  Other, please specify:…………………………………..

Email: …………………………………………Telephone/Mobile number: ……………………...

Number of household member:………persons

Number of working member:….., those are  Father  Mother  Other, specify……….

Number of dependent member:…………………………………………………………………

Monthly family income (in Vietnam Dong):…………………………………………….(VND)

**Parent details**

**Mother**

Full name:

DOB (DD/MM/YYYY): _ _ /_ _ / _ _ _ _

Mother’s educational level at the time of child’s birth:  Illiterate  Primary school completed  Secondary school completed  High school completed  University/College graduation  Post-graduation (please specify)…………………………………………………..…………….

Other (please specify)…………………………………………………………………………….

Mother’s occupation at the time of child’s birth

☐ Manager, professional, self-employed ☐ Office worker ☐ Skilled manual worker
☐ Unskilled/manual labourer ☐ Military or armed forces personnel
☐ Unemployed ☐ Other (please specify): ___________

Current occupation of mother:
☐ Manager, professional, self-employed ☐ Office worker ☐ Skilled manual worker
☐ Unskilled/manual labourer ☐ Military or armed forces personnel
☐ Unemployed ☐ Other (please specify): ___________

**Father**

Full name:

DOB (DD/MM/YYYY): _ _ /_ _ / _ _ _ _

Father’s educational level at the time of child’s birth:  Illiterate  Primary school completed  Secondary school completed  High school completed  University/College graduation  Post-graduation (please specify)…………………………………………………..…………….

Other (please specify)…………………………………………………………………………….

Father’s occupation at the time of child’s birth

☐ Manager, professional, self-employed ☐ Office worker ☐ Skilled manual worker
☐ Unskilled/manual labourer ☐ Military or armed forces personnel
☐ Unemployed ☐ Other (please specify): ___________

Current occupation of Father:
☐ Manager, professional, self-employed ☐ Office worker ☐ Skilled manual worker
☐ Unskilled/manual labourer ☐ Military or armed forces personnel
☐ Unemployed ☐ Other (please specify): ___________

Are the parents biologically related (within three generations)?

☐ Yes (please specify: ……………………………)    ☐ No

Is there any other family member with disability?  Yes  No

If Yes, how the other disabled family members related to the child with CP?

 Sibling  Parent Other, please specify …….........................................................

Please specify the disability/impairment of the other family member(s):………………….

**Birth details of the child with CP and maternal pregnancy details**

Birth place:

 Hospital  Commune health station ☐ Maternity home  Outside a health facility

If outside a health facility,  Unplanned  Planned

If planned, delivery attended by  Skilled birth attendant/TBA

 Other family member  Doctor/Midwife

Birth weight (in gram):……………. or  Unknown Born at:……………. week gestation

Was there any sign of birth asphyxia (e.g., requiring resuscitation, very weak breathing/cry, cyanosis, bradycardia, poor muscle tone/reflexes, meconium strained liquor, seizure – circle which applies):

 Yes, please specify………………………………………………...  No  Don’t know

Neonatal jaundice  No  Yes, physiological jaundice (appeared after the first 24 hours after birth)

 Yes, pathological jaundice (appeared within the first 24 hours after birth)  Don’t know

Did the child show any early feeding difficulties (first month of life) manifesting as poor sucking abilities?

 Yes, please specify………………………………………………….  No  Don’t know

Mode of delivery:  Spontaneous vaginal delivery  Instrumental delivery  Caesarean section

If Caesarean section:  Planned  Emergency section

Any complications leading up to and during child birth/labour?  Yes  No

If Yes:  Obstructed/prolonged labour (active phase of labour >12 hours)

 Malpresentation  Pre-eclampsia/Eclampsia  Haemorrhage  Premature rupture of membranes/premature labour (<37 weeks)  Other, please specify:………………….

Mother had fever before labor?  No   Yes   Don’t know

If fever, was it accompanied by rash?  No   Yes   Don’t know

If rash, specify the time of onset:

 Pregnancy 1–12 weeks  Pregnancy 13–28 weeks  Pregnancy 29–40 weeks  Don’t know

Did the Mother experience any febrile illness during labour/child birth?

 Yes (when……………………)  No  Don’t know

Did the Mother receive any antenatal care during pregnancy?  Yes  No

If Yes,  Regular  Irregular (but > 2 visits)  Irregular ($\leq$ 2 visits)

Did the Mother experience any febrile illness during pregnancy?  Yes  No  Don’t know

If Yes, was it associated with rash?  Yes  No  Don’t know

If Yes, please specify when did the rash appear?

 1-12 weeks  13-28 weeks  29-40 weeks  Don’t know

Did the Mother receive any nutritional supplementation during pregnancy (e.g., Iron, Folic acid)?

 Yes  No  Don’t know

Hospital of neonatal transfer (if applicable, specify the highest level):…………………………

Receive more than routine care?  Yes – NICU  Yes, please specify………………...  No

If Yes, total length of stay………. days.

Was MRI completed?  Yes (when – child’s age………………………………………)  No

MRI results:………………………………………………………………………………………...

………………………………………………………………………………………………………

Was this a multiple birth?  Yes (  Twins  Triplets  4  5  6  >6)  No

Birth order of child with CP in the family:…………………………………………………………

Was there any assistance with conception?  No  Yes, type unknown

 Yes, if known please choose which type of assistance:  Fertility drugs  Ovulation stimulation only  Artificial insemination  ICSI  IVF  GIFT Other  Not stated

Number of previous live births to Mother:………………………………………………………. Number of previous stillbirths (> 20 week gestation):……………………………………………..

Number of previous miscarriage: (<20 week gestation):…………………………………………...

**Details of the child’s cerebral palsy condition** (Note: If you are unsure about any detail, please leave it blank)

Age at which movement disorder was first noted by a doctor: …… years …… months

Age at confirmed diagnosis of cerebral palsy: …… years …… months

| **Timing of CP** |  Unknown |  During pregnancy and up to first 28 days of life (pre & perinatal) |  After first 28 days of life (postnatal) |
| --- | --- | --- | --- |
| **Causes of CP** |  |  Unknown   Microcephaly   Poor fetal growth or intrauterine growth restriction (IUGR)   In utero cytomegalovirus   Toxoplasmosis, rubella, herpes   Encephalopathy   Meningitis   Encephalitis   Other infection (please list in the comments)   Other (please list in the comments) | **Head injury**   Motor vehicle accident   Non accidental   Fall   Other (please describe in the comments)  **Infection**   Unspecified cause   Bacterial   Dehydration due to gastroenteritis  **Stroke or CVA**   During or following surgical procedure   Spontaneous   Associated with other cardiac complications  **Other**   Post seizure   Near sudden infant death syndrome (SIDS)   Post immunization   Near drowning   Peri-operative hypoxia   Apparent life-threatening event   Other (please describe in the comments) |
|  |  |  |  |

Comments:

**Immunization history**

Fully immunized?  Yes  No

 *BCG vaccine after birth*

 *HepB vaccine after birth*

*DPT-HepB-Hib and OPV (1^st^, 2^nd^, 3^rd^ doses) at*  *2 months,*  *3 months,*  *4 months*

 *Polio vaccine at 5 months (4^th^ dose)*

 *Measles at 9 months*

*Measles and Rubella at 18 months*

 *Diphtheria-Pertussis-Tetanus-Hib 4^th^ dose vaccine at 18 months*

*Japanese encephalitis vaccine at 12 months:*  *1^st^ dose at 12 months,*  *2^nd^ dose after 1^st^ dose 1-2 weeks*  *3^rd^ dose after 2^nd^ dose 1 year*

If no, reason why the child missed immunization?

 Immunization is not important  Immunization is not safe

 Child is refused vaccination  Health problem

 Parents are reluctant to do  Transport problem  Other (please specify……………….…)

Is there any BCG mark?  Yes  No

**Education (children ≥ 6 years old)**

Is the child currently attending mainstream school?  Yes  No

Type of school:  Primary  Secondary  High school  Other, specify………….

Is the child currently attending any special school?  Yes  No

If not attending any school (>6 years), reason why?

 Working  School too far  Disability not accepted by school  Lack of money  Parents refused.

 Other (please specify:…………………………………………………………………...)

**Rehabilitation**

Has the child ever received any rehabilitation service or other related support?  Yes  No

If yes, what type of support was received?

 Assistive/Adaptive device  Surgery  Therapy exercises  Advice

 Other, please specify…..…............................................................................................................

What type of location for accessing these rehabilitation services?

 Home based  NGO center  Hospital  Private clinic

 Other, please specify…………………………………………………………………………….

Age when the child first received any rehabilitative services:…………….(in years)

Frequency of rehabilitation therapy in the past year:

☐ Daily   ☐ 1–2 days/week   ☐ 1–2 days/month   ☐ Less than 12 days/year   ☐ Not receiving therapy

Reason why the child never received rehabilitation?

 Not aware  No money  Transport problem  Other, please specify……………………..

**General health**

Number of hospitalizations in the past 6 months: ........... times.

Frequently encountered respiratory problems:  No  Aspiration pneumonia   Bronchitis

 Other, please specify:…………………………………………………………………………….

Frequently encountered musculoskeletal problem:  No  Hip dislocation  Scoliosis

 Contraction  Unknown  Other, specify:………………………………………

*The above information has been collected on: _ _ /_ _ / _ _ _ _*

**Health Professional details (Register staff complete)**

Full name:…………………………………………………………………………………………..

Occupation:  Medical Doctor  Rehabilitation Doctor  Physiotherapist  Other, specify

Place of work:………………………………………………………………………………………

Email:…………………………………………….. Telephone/Mobile number:…………………..

**Additional questions suggested by CPFAV**

**Sleep disorders**

| In the past month, has the child had difficulty falling asleep? |  Never |  Sometimes |  Often |  Every night |
| --- | --- | --- | --- | --- |
| In the past month, has the child frequently woken up during the night? |  Never |  Sometimes |  Often |  Every night |
| On average, how long does it take the child to fall asleep each night? |  <5 minutes |  5-15 minutes |  16-30 minutes |  >30 minutes |
| Does the child use sleep medication? |  Never |  Sometimes |  Often |  Every night |
| How would you rate the child’s sleep quality over the past month? |  Excellent |  Good |  Normal |  Bad |
| On average, how many times does the child wake up each night? |  0 times |  1-2 times |  3-5 times | >5 times |
| In the past month, how many hours of actual sleep does the child get each night on average? |  | | | |

**Constipation problems**

| Bowel movements |  Unsure |  ≥ 4 times a week |  ≤ 3 times a week |  Other, specify |
| --- | --- | --- | --- | --- |
| Difficulties when the child has a bowel movement: straining, pain, crying, anal bleeding |  Never |  Sometimes |  Often |  Always |
| The child uses enemas or manual assistance |  Never |  Sometimes |  Often |  Always |
| Stools are hard, dry, or scattered in lumps |  Never |  Sometimes |  Often |  Always |

**Controlling behaviours**

| Behaviors | Never | Sometimes | Often | Usually |
| --- | --- | --- | --- | --- |
| Crying, screaming, easily irritable for unknown reasons |  |  |  |  |
| Throwing objects, destroying furniture |  |  |  |  |
| Hitting others, biting, pinching |  |  |  |  |
| Self-harming |  |  |  |  |
| Avoiding social activities |  |  |  |  |
| Difficulty concentrating, easily distracted |  |  |  |  |
| Engaging in repetitive behaviors |  |  |  |  |
| Difficulty controlling emotions |  |  |  |  |

**Vietnam Cerebral Palsy Register form (PART B)**

**General information**

Full name:.………………………………………………………………………………………….

Gender:  Male  Female DOB (DD/MM/YYYY): _ _ /_ _ / _ _ _ _

Address:…………………………………………………………………………………………….

House no.:.…………….. Street name:.…………………………District:.……………………….

Province:.………………………………………………………………………………………......

Name of parent/ main caregiver: ………………………………………………………………….

Email: …………………………………………Telephone/Mobile number: ……………………...

| **Type of CP** | **Main type at initial diagnosis** | **Main type at or over age 5** | **Secondary type at or over age 5** |
| --- | --- | --- | --- |
| **Spasticity** |  |  |  |
| Left hemiplegia/Monoplegia |  |  |  |
| Right hemiplegia/Monoplegia |  |  |  |
| Diplegia |  |  |  |
| Triplegia |  |  |  |
| Quadriplegia |  |  |  |
| **Dyskinesia** |  |  |  |
| Mainly athetosis |  |  |  |
| Mainly dystonia |  |  |  |
| **Ataxia** |  |  |  |
| **Hypotonia** |  |  |  |
| **Resolved by age of 5** |  |  |  |
| **Known syndrome – not CP** |  |  |  |
| **Unknown** |  |  |  |

| **Severity of CP**  (please see GMFCS sheet for further information) | **Initial diagnosis** | **At or over age 5** |
| --- | --- | --- |
| GMFCS level I |  |  |
| GMFCS level II |  |  |
| GMFCS level III |  |  |
| GMFCS level IV |  |  |
| GMFCS level IV |  |  |

Comment (e.g., any difficulty in assessing the GMFCS level):……………………………………

Ability to handle objects in daily life (please tick one, please see MACS/Mini MACS sheet for further information)

 Level I  Level II  Level III  Level IV  Level V

Communication function (CFCS) with child at or over age 4 (please tick one, please see CFCS sheet for further information)

CFCS level I CFCS level II CFCS level III CFCS level IV CFCS level V

Were there any birth defects present? (e.g. Congenital heart defect):

 No  Yes, please specify………………………………………………………………….

Is there a known syndrome?  No  Yes, please specify………………………………….........

Presence of associated impairments (at or over age 5)

***Epilepsy***  No  Yes  Resolved by age 5  Unknown

***Intellectual***  No impairment  Mild

 Probably no impairment  Moderate

 Probably some impairment  Severe

 Unknown

***Speech***   No impairment  Non verbal

 Some impairment  Unknown

VIKING Speech Scale (at or over age 4)

VIKING level I VIKING level II VIKING level III VIKING level IV

***Visual***   No impairment  Functionally blind

 Some impairment (e.g., glasses)  Unknown

Strabismus  No  Yes  Unknown

***Hearing***  No impairment Bilateral deafness

 Some impairment (includes conductive hearing loss)  Unknown

**Nutrition**

Current weight of the child:………..Kg Head circumference of the child:…………cm

Current height of the child:………..cm Mid-upper arm circumference:…………cm

Knee height of the child:………..cm

Digestive/Nutrition problems:

 No  Growth delay  Difficulty in swallowing  Constipation

 Gastroesophageal Reflux Disease (GERD)  Dental issues

 Other, please specify:……………………..……………………………………………………...

Eating and Drinking Ability Classification System (EDACS) at or over age 3:

 Level I  Level II  Level III  Level IV  Level V

Level of support with EDACS ay or over age 3:

 Independent Need support Totally dependent

Any associated congenital anomalies? (e.g., congenital heart disease)
☐ No   ☐ Yes, please specify: ……………………….

Any known clinical syndromes? ☐ No   ☐ Yes, please specify: ……………………….

**Other comments:** ……………………………………………………………………………………

……………………………………………………………………………………………………………………………………………………………………………………………………………………

**Health Professional details (Register staff complete)**

Full name:…………………………………………………………………………………………..

Occupation:  Medical Doctor  Rehabilitation Doctor  Physiotherapist  Other, specify

Place of work:………………………………………………………………………………………

Email:…………………………………………….. Telephone/Mobile number:…………………..
