# Supplementary material for: Vietnam Cerebral Palsy Register: protocol for co-design of a national register with people with lived experience of cerebral palsy
Source: BMJ Open. 2026 Jul 30;16(7):e116376. doi: 10.1136/bmjopen-2026-116376 (PMC13423169; doi:10.1136/bmjopen-2026-116376)
Supplement: online supplemental file 2 [file bmjopen-16-7-s002.docx]

# **Supplementary file 2.**

# Vaccine-preventable diseases and mandatory vaccines included in the Expanded Programme on Immunization in Vietnam (Circular 38/2017/TT-BYT Introducing lists of infectious diseases, scope and recipients of compulsory vaccines and biologicals)

|  | **Vaccine-preventable disease in Vietnam** | **Vaccine** | **Target population** | **Immunization schedule** |
| --- | --- | --- | --- | --- |
| 1 | Hepatitis B | Monovalent Hepatitis B vaccine | Newborn infants | Birth dose administered within 24 hours after birth |
|  |  | Combination vaccine containing Hepatitis B component | Children aged <1 year | Dose 1: 2 months of age;  Dose 2: at least 1 month after Dose 1;  Dose 3: at least 1 month after Dose 2 |
| 2 | Tuberculosis | BCG vaccine | Children aged <1 year | Single dose administered within 1 month after birth |
| 3 | Diphtheria | Combination vaccine containing diphtheria component | Children aged <1 year | Dose 1: 2 months of age;  Dose 2: at least 1 month after Dose 1;  Dose 3: at least 1 month after Dose 2 |
|  |  |  | Children aged <2 years | Booster dose at 18 months of age |
| 4 | Pertussis | Combination vaccine containing pertussis component | Children aged <1 year | Dose 1: 2 months of age;  Dose 2: at least 1 month after Dose 1;  Dose 3: at least 1 month after Dose 2 |
|  |  |  | Children aged <2 years | Booster dose at 18 months of age |
| 5 | Tetanus | Combination vaccine containing tetanus component | Children aged <1 year | Dose 1: 2 months of age;  Dose 2: at least 1 month after Dose 1;  Dose 3: at least 1 month after Dose 2 |
|  |  |  | Children aged <2 years | Booster dose at 18 months of age |
|  |  | Monovalent tetanus vaccine | Pregnant women | **For women with no documented history of tetanus vaccination or who have received fewer than three doses:**  Dose 1: as early as possible during the first pregnancy;  Dose 2: at least 1 month after Dose 1;  Dose 3: at least 6 months after Dose 2 or during a subsequent pregnancy;  Dose 4: at least 1 year after Dose 3 or during a subsequent pregnancy;  Dose 5: at least 1 year after Dose 4 or during a subsequent pregnancy. |
|  |  |  |  | **For women who have previously received three primary doses of a tetanus-containing vaccine:**  Dose 1: as early as possible during the first pregnancy;  Dose 2: at least 1 month after Dose 1; Dose 3: at least 1 year after Dose 2. |
|  |  |  |  | **For women who have previously received three primary doses and one booster dose of a tetanus-containing vaccine:**  Dose 1: as early as possible during the first pregnancy;  Dose 2: at least 1 year after Dose 1. |
| 6 | Poliomyelitis | Oral polio vaccine (OPV) | Children aged <1 year | Dose 1: 2 months of age;  Dose 2: at least 1 month after Dose 1;  Dose 3: at least 1 month after Dose 2 |
|  |  | Inactivated polio vaccine (IPV) | Children aged <1 year | One dose at 5 months of age |
| 7 | Haemophilus influenzae type b disease | Monovalent Hib vaccine or combination vaccine containing Hib component | Children aged <1 year | Dose 1: 2 months of age;  Dose 2: at least 1 month after Dose 1;  Dose 3: at least 1 month after Dose 2 |
| 8 | Measles | Monovalent measles vaccine | Children aged <1 year | One dose at 9 months of age |
|  |  | Combination vaccine containing measles component | Children aged <2 years | One dose at 18 months of age |
| 9 | Japanese Encephalitis | Japanese Encephalitis vaccine | Children aged 1-5 years | Dose 1: 12 months of age;  Dose 2: 1-2 weeks after Dose 1;  Dose 3: 1 year after Dose 2 |
| 10 | Rubella | Combination vaccine containing rubella component | Children aged <2 years | One dose at 18 months of age |
