# Supplementary material for: Vietnam Cerebral Palsy Register: protocol for co-design of a national register with people with lived experience of cerebral palsy
Source: BMJ Open. 2026 Jul 30;16(7):e116376. doi: 10.1136/bmjopen-2026-116376 (PMC13423169; doi:10.1136/bmjopen-2026-116376)
Supplement: Supplementary file 3 [file bmjopen-2026-116376.reviewer_comments.pdf]

PEER REVIEW HISTORY

BMJ Open publishes all reviews undertaken for accepted manuscripts. Reviewers are asked to complete a checklist review form and are provided with free text boxes to elaborate on their assessment. These free text comments are reproduced below.

ARTICLE DETAILS

Title (Provisional)

The Vietnam Cerebral Palsy Register: Protocol for co-design of a national register with people with lived experience of cerebral palsy

Authors

Khuc, Thi Hong Hanh; Cao, Minh Chau; Karim, Tasneem; Dinh, Thi Lan Anh; Nguyen, Thi Huong Giang; Nguyen, Thi Van Anh; Pham, Minh Chau; Nguyen, Van Bang; Rawal, Lal; Mcintyre, Sarah; Khandaker, Gulam; Elliott, Elizabeth Jane; Vietnam, Cerebral Palsy Family Association

---

VERSION 1 - REVIEW

**Reviewer**	**1**
**Name**	**Chen, Brian Po-Jung**
**Affiliation**	**Chang Gung Memorial Hospital Linkou**
**Date**	**22-May-2026**
**COI**	**None**

Thank you for the opportunity to review this study protocol. I appreciate the authors' effort in providing a thorough review of the existing literature and the history of cerebral palsy registries. The Introduction section effectively reinforces and clarifies the rationale for conducting the proposed study. Overall, the manuscript clearly articulates the protocol and provides a comprehensive overview of the key aspects related to establishing a CP registry in a Southeast Asian society, specifically in Vietnam.

That said, I have a few comments on areas that may benefit from further clarification and minor formatting adjustments. I would be happy to continue the discussion with the authors during the revision process to help further refine the manuscript and support its successful completion

---

Comment 01

Page 3; Line 41-42 (Abstract)

"The VCPR will contribute data to the Global LMIC CP Register."

Though it's the text within the abstract, it would be nice to state the name of the register in full as "Global Low and Middle Income Cerebral Palsy Register."

If the word count (< 300 words) is a concern for the authors, removing abbreviations that are not used repeatedly can be helpful, e.g., "(NCH)" on Line 23, "(GMFCS)" on Line 34, "(MACS)" on Line 36. Also, on Line 36-37, the examples of associated impairments "(e.g. vision, hearing, intellectual disabilities)" can be removed if the authors agree.

Moreover, throughout the protocol, the abbreviation for "Global Low and Middle Income Cerebral Palsy Register" is written inconsistently as either "GLM CPR" without a hyphen or "GLM-CPR" with a hyphen. Although this may be a minor issue, it would be helpful to use a consistent abbreviation throughout the manuscript.

Comment 02

Page 3; Line 46 (Abstract)

"Ethics approval has been obtained from Vietnamese and international organizations."

It is not entirely clear to me what the "international organizations" mentioned in this sentence refer to (I suspect it might refer to any Australian institutions?). Since this protocol aims to establish the VCPR, could you clarify the role these specific international organizations would play?

Comment 03

Page 6; Line 6-8 (Introduction)

"Full vaccination coverage was lower among children with CP (82.7%) than in children in the general population (96.4%)."

As noted in the authors' previous study (Khuc et al., 2025), *"The major factors influencing the lower rates of immunization in CP included: younger age, living in an earth/sand house, being born at home, having a mother with a low level of education, age at CP diagnosis under three years old, bilateral involvement, associated impairments (i.e., epilepsy, intellectual, visual, and speech impairments), GMFCS Levels IV–V, and undernutrition."* and in addition, as mentioned in Lines 40–42 on Page 5, *"…registers in LMIC often additionally organize or provide services and supports…"*, I understand that the immunization history may have been assessed according to the national public health vaccination schedule. However, even among children with CP in LMICs who are eventually fully vaccinated, some may still have received their vaccinations later than the recommended age. Therefore, if available, would the authors

consider documenting the actual age at which each vaccination was received? This information may help further clarify whether delayed vaccination, rather than only incomplete vaccination, contributed to the observed findings.

Comment 04

Page 6; Line 33-36 (Introduction)

"In our literature review (unpublished), we found that all published studies on children with CP in Vietnam were hospital-based and that no community-based research has been conducted."

This is indeed a critical point for a newly formed register. How do the authors prevent the fate of the VCPR ends up with the hospital-based children with CP? Besides CPFAV, are there any other national or governmental societies that may assist to achieve this ambition.

Comment 05

Page 6; Line 46-48 (Introduction)

"CPFAV had approximately 10,000 members, including 4368 children (aged < 18 years) and 347 adults (aged ≥ 18 years) living with CP and their families."

Age distribution remains a major challenge in both CP healthcare and CP registries across countries. I understand that this may partly reflect the relatively recent establishment of CPFAV in late 2017. However, do the authors have any further insight into why adults with CP are proportionally underrepresented compared with children in CPFAV? Understanding this issue and developing reasonable strategic plans may help sustain the development of the VCPR and support the long-term sustainability of this upcoming registry, particularly because follow-up may be limited for mature ambulatory individuals with CP who have relatively less active medical issues.

Comment 06

Page 7; Line 2 (Introduction)

"... modeled on the ACPR and the GLM-CPR."

I don't believe the full term of GLM-CPR, Global Low and Middle Income Cerebral Palsy Register, has been mentioned before here, except in the Abstract section.

Comment 07

Page 7; Line 29-34 (Introduction)

"The VCPR is a partnership between Phenikaa University Hanoi, the Cerebral Palsy Family Association of Vietnam, the National Children's Hospital Hanoi and the University of Sydney. Collaborators include the Cerebral Palsy Alliance Australia, Central Queensland University,

Hanoi Medical University and the Global LMIC CP Register."

I understand that the authors may wish to provide the full name of each party in this paragraph and I personally am not against this at all. However, to improve readability, the authors may consider listing each full name followed by its abbreviation in parentheses, particularly for parties that are mentioned elsewhere in the manuscript, for instance:
"The Vietnam Cerebral Palsy Register (VCPR) is a partnership between Phenikaa University Hanoi, the Cerebral Palsy Family Association of Vietnam (CPFAV), the National Children's Hospital Hanoi (NCH Hanoi) and the University of Sydney. Collaborators include the Cerebral Palsy Alliance Australia, Central Queensland University, Hanoi Medical University and the Global Low and Middle Income Cerebral Palsy Register (GLM-CPR)."

Comment 08

Page 7; Line 36-41 (Introduction)

"Through our shared vision for optimal outcomes for people living with CP in Vietnam, our team of clinicians and researchers has established a strong relationship with CPFAV, including with Ms Dinh Thi Lan Anh, the CEO and founder of CPFAV, who was inspired by her journey with her daughter with CP to establish CPFAV. She will represent CPFAV in the partnership, including as a member of the CPVR Advisory Board."

I wonder whether this paragraph would fit better in the Acknowledgments section rather than here at the end of the Introduction. Although the content itself seems appropriate, it appears somewhat disconnected from the surrounding context in the Introduction. Therefore, the authors may consider keeping the paragraph unchanged but relocating it to the Acknowledgments section.

Comment 09

Page 8; Line 40-42 (Methods: Register design and settings)

"... children with CP aged <18 years..."

Is there a specific reason why only children younger than 18 years will be included in the future VCPR? What are the major challenges or concerns associated with including individuals with CP who are 18 years of age or older?

Does the statement made later in the "Inclusion/exclusion criteria" section help explain the rationale behind this age restriction? Even so, would including individuals with CP older than 18 years potentially raise any additional issues?

Comment 10

Page 9; Line 22-27 (Methods: Inclusion/exclusion criteria)

"Any child… will be eligible for inclusion."

One major concern regarding the use of the aforementioned definitions or elements as inclusion/exclusion criteria, particularly for retrospectively acquired data, is that these criteria may rely on descriptions of past events in specific individuals. To avoid potential contamination or misclassification in data inclusion, do the authors rely on other documentation, such as medical records from reliable sources or national health databases, to retrospectively confirm the diagnosis of each individual and maintain the accuracy of the registry? If so, it would be helpful to elaborate on how this has been, or will be, implemented during the registry development process.

Comment 11

Page 10; Line 11-38 (Table 1)

"Table 1. Children with CP registered in 63 CFPAV hubs in 34 provinces (N= 4368; Updated 25/12/2025)"

1. The abbreviation is mis-spell as "CFPAV" instead of the correct "CPFAV."
2. The table is presented in ascending order according to the number of children available in each hub. What is the primary known reason for the difference in sample size (from 718 to 6) across hubs? Is it related to the service capacity of each facility, the population size of the catchment area, or the socioeconomic status of the region?

Comment 12

Page 11; Line 26 (#3)

"The VCPR may contribute selected de-identified data to the Global LMIC CP Register…"

Which specific data elements will be selected for contribution to the GLM-CPR?

Comment 13

Page 12-13 (Figure 1)

Figure 1 is inserted here but is not mentioned anywhere in the main text. Could the authors add a reference to Figure 1 in the relevant section and clarify what this figure is intended to illustrate?

Comment 14

Page 14; Line 46 (Data collection)

"For use by the VCPR, this form has been updated…"

It would be helpful if the authors could further specify what has been updated and explain the rationale for these updates. This would help readers better understand the fundamental differences between Vietnam and other countries with established CP registries.

Comment 15

Page 14; Line 17-34 (Data collection)

"All caregivers of children… and other test results."

This is a critical yet challenging process in establishing the registry. At this stage, do the authors have any estimates of the expected percentage of successful data collection at each stage for Part A and Part B? Alternatively, have the authors set a minimum target percentage of data completion that must be reached for the identified individuals at each stage?

Comment 16

Page 14; Line 44-47 (Data collection)

"All data will be collected by qualified and trained doctors, physiotherapists and caregivers with a background in health, or qualified research assistants. Data collected will be reviewed weekly by senior investigators to ensure completeness and accuracy."

Could the authors further elaborate on why caregivers with a background in health will be included in the data collection process? What types of professional backgrounds or specialties would be considered acceptable qualifications for a data collector?

In addition, are the "qualified and trained doctors, physiotherapists, caregivers with a background in health, or qualified research assistants" mentioned here the same as the data collectors described later on Page 16?

Comment 17

Page 14; Line 48-50 (Data collection)

"For children enrolled in the VCPR before the age of five years..."

What is the purpose of re-contacting families when the child reaches five years of age? In addition to this five-year follow-up, do the authors expect to re-contact the family at any later stage of the individual's life?

Comment 18

Page 15; Line 43-44 (Variables: Associated features)

"... growth and nutritional status (Z-scores and percentiles for current weight for age, height for age,..."

The authors do not, and certainly should not, restrict inclusion in the VCPR based on GMFCS level. However, it is well recognized that, in children with CP classified as GMFCS Levels III–V, accurate assessment of growth based on standing body height or arm span can be challenging

because of moderate to severe skeletal deformities involving the lower limbs and spine. Would the authors consider including additional anthropometric measurements that may better reflect actual growth status in this population?

Comment 19

Page 15; Line 43-44 (Variables: vaccination status)

"... vaccination status (fully vaccinated or not according to the EPI for Vietnam), receipt of specific vaccines, reason for missed vaccines, presence of BCG scar."

Each country may have its own vaccination regulations and schedules, depending on geographic location, public health policy, and historical context. It may be helpful for the authors to provide appendix information on the current vaccination regulations in Vietnam, including vaccine types and recommended timetables, to help future readers better interpret the results.

Comment 20

Page 16; Line 10-17 (Data collectors)

"Training of data collectors"

I assume that the recruited data collectors will be responsible for collecting data from NCH. Will they also be involved in data collection on the CPFAV side? Based on the current plan, how many data collectors are expected to be required to manage the workload of this project?

Comment 21

Page 19; Line 10-17 (Data entry)

"... will be entered into the CPRV database…"

This is the only instance in which the abbreviation "CPRV" is used throughout the protocol. Could this be a typographical error, and should it instead refer to "VCPR"?

Page 8; Line 41

"... CPVR Advisory Board."

Page 19; Line 46-47

"We will regularly report the results of CPVR data analysis…"

Same as above, should these two abbreviations be changed to "VCPR" instead?

Comment 22

Page 17; Line 12 (Expert Advisory and Investigative Team)

"... The Vietnam Cerebral Palsy Register (VCPR) will be led by…"

The full form of VCPR may not be necessary here. Using the abbreviation alone should be sufficient: "The VCPR will be led by…"

Comment 23

Page 4; Line 20-22 (Strength and limitations of this study)

"Potential selection bias, with under-representation of children not accessing hospitals or CPFAV networks."

Although this potential limitation has already been noted as the only one in this section on Page 4, it would be helpful if the authors could include a separate "Limitations and Challenges" section near the end of the protocol, possibly after Dissemination on Page 17 and before Authors' Contributions on Page 18. This section could further discuss known limitations related to the current study design, the restriction from the Vietnamese clinical and research environment, and any anticipated challenges in establishing the VCPR.

VERSION 1 - AUTHOR RESPONSE

Response to reviewers

**Comment 01 (Page 3; Line 41-42 - Abstract):**<br><br>"The VCPR will contribute data to the Global LMIC CP Register."<br><br>Though it's the text within the abstract, it would be nice to state the name of the register in full as "Global Low and Middle Income Cerebral Palsy Register."<br><br>If the word count (<300 words) is a concern for the authors, removing abbreviations that are not used repeatedly can be helpful, e.g., "(NCH)" on Line 23, "(GMFCS)" on Line 34, "(MACS)" on Line 36. Also, on Line 36-37, the examples of associated impairments "(e.g. vision, hearing, intellectual disabilities)" can be removed if the authors agree.<br><br>Moreover, throughout the protocol, the abbreviation for "Global Low and Middle Income Cerebral Palsy Register" is written	Thank you for these helpful suggestions.<br><br>We have revised the manuscript accordingly. "Global Low-and Middle-Income Country Cerebral Palsy Register" has been written in full in the abstract and the abbreviation "GLM CPR" is used consistently throughout the manuscript.<br><br>Unnecessary abbreviations have been removed where appropriate. However, the abbreviation "NCH" has been retained because it appears more than once in the abstract. We prefer to keep the examples of associated impairments to provide readers with a clearer understanding of the types of impairments that have been captured in this study.<br><br>Please see comment above.

inconsistently as either "GLM CPR" without a hyphen or "GLM-CPR" with a hyphen.<br><br>Although this may be a minor issue, it would be helpful to use a consistent abbreviation throughout the manuscript.	
**Comment 02 (Page 3; Line 46 - Abstract):**<br><br>"Ethics approval has been obtained from Vietnamese and international organizations."<br><br>It is not entirely clear to me what the "international organizations" mentioned in this sentence refer to (I suspect it might refer to any Australian institutions?).<br><br>Since this protocol aims to establish the VCPR, could you clarify the role these specific international organizations would play?	Thank you for this comment.<br><br>We have clarified in the main body of the text that the "international organizations" referred to are CQUniversity and the University of Sydney, Australia, where the Vietnamese organizations who provided ethics approval are also listed in full.<br><br>"Ethics approval for hospital-based surveillance (the existing data of VCPR) was obtained from the University of Sydney Human Research Ethics Committee (HREC) (2016/456) (approval remains active), Hanoi Medical University (1722/QD-DHYHN) and the NCH (812/QD-BVNTU). Ethics approval for VCPR data collection in partnership with CPFAV and community-based surveillance was obtained from Phenikaa University, Vietnam (No: 024-03.02/DHP-HDDD). Ethics approval was endorsed by Central Queensland University HREC (No: 0000025568)." (line 470 - 476)<br><br>The following text in the abstract has been revised and the role of international institutions has been clarified as below:<br><br>"Ethics approval has been obtained from Vietnamese and Australian institutions, whose role, is to provide ethical oversight and research governance for the PhD project supporting the VCPR." (line 51 - 53)
**Comment 03 (Page 6; Line 6-8 - Introduction):**<br><br>"Full vaccination coverage was lower among children with CP (82.7%) than in children in the general population (96.4%)."<br><br>As noted in the authors' previous study (Khuc et al., 2025), "The major factors influencing the lower rates of immunization in CP included: younger age, living in an earth/sand house, being born at home, having a mother	Thank you for your constructive comment.<br><br>We agree that information on vaccination timing could provide additional insights into delayed vaccination.<br><br>However, to maintain the feasibility of the register and minimise participant burden, the VCPR will collect only age-appropriate vaccination status at the time of data collection

with a low level of education, age at CP diagnosis under three years old, bilateral involvement, associated impairments (i.e., epilepsy, intellectual, visual, and speech impairments), GMFCS Levels IV-V, and undernutrition."<br><br>and in addition, as mentioned in Lines 40-42 on Page 5, "...registers in LMIC often additionally organize or provide services and supports...", I understand that the immunization history may have been assessed according to the national public health vaccination schedule.<br><br>However, even among children with CP in LMICs who are eventually fully vaccinated, some may still have received their vaccinations later than the recommended age.<br><br>Therefore, if available, would the authors consider documenting the actual age at which each vaccination was received?<br><br>This information may help further clarify whether delayed vaccination, rather than only incomplete vaccination, contributed to the observed findings.	rather than the dates of individual vaccine doses. This approach is consistent with other cerebral palsy registers globally, supporting data harmonisation and enabling comparison across geographic settings.<br><br>While we recognise the value of collecting more detailed vaccination data, including individual vaccination dates, this is beyond the current scope of the register. The VCPR data will help identify priorities for future research, which may involve more detailed investigation of specific subgroups and areas of interest.
**Comment 04 (Page 6; Line 33-36 - Introduction):**<br><br>"In our literature review (unpublished), we found that all published studies on children with CP in Vietnam were hospital-based and that no community-based research has been conducted."<br><br>This is indeed a critical point for a newly formed register.<br><br>How do the authors prevent the fate of the VCPR ends up with the hospital-based children with CP?<br><br>Besides CPFAV, are there any other national or governmental societies that may assist to achieve this ambition.	Thank you for this important comment. We agree that ensuring representation beyond hospital-based populations is a key challenge for any newly established CP register.<br><br>The VCPR will incorporate both healthcare-based and community-based recruitment pathways. In addition to recruitment through healthcare facilities, the VCPR will recruit through the CPFAV, with which it is being developed in partnership. CPFAV is the only national network of people with cerebral palsy and their families, with 63 hubs spanning all provinces of Vietnam. The VCPR website will provide opportunities for children with CP and their families attending these hubs to register directly.<br><br>In the Methods section we describe that long-term recruitment will involve a multicentre approach across healthcare facilities and

	associations of people with disabilities at different levels of the health system, together with community-based identification of children who newly join CPFAV as below:<br><br>"In the longer term, data will be collected prospectively through a multicentre recruitment approach involving healthcare facilities, associations of people with disabilities at different levels of the health system across Vietnam, as well as through community-based identification of children who newly join the CPFAV. Healthcare facilities, associations of people with disabilities nationwide will be invited to participate in the VCPR either as data collection hubs or by referring children newly diagnosed with CP and their families for enrolment." (line 277 - 282)
**Comment 05 (Page 6; Line 46-48 - Introduction):**<br><br>"CPFAV had approximately 10,000 members, including 4368 children (aged < 18 years) and 347 adults (aged ≥ 18 years) living with CP and their families."<br><br>Age distribution remains a major challenge in both CP healthcare and CP registries across countries.<br><br>I understand that this may partly reflect the relatively recent establishment of CPFAV in late 2017. However, do the authors have any further insight into why adults with CP are proportionally underrepresented compared with children in CPFAV?<br><br>Understanding this issue and developing reasonable strategic plans may help sustain the development of the VCPR and support the long-term sustainability of this upcoming registry, particularly because follow-up may be limited for mature ambulatory individuals with CP who have relatively less active medical issues.	Thank you for this important observation.<br><br>The relatively small number of adults with CP known to CPFAV likely reflects the organisation's primary focus on supporting Vietnamese children with CP and their families since its establishment, facilitating access to rehabilitation, and informing policy development. Consistent with the GLM CPR and other CP registers in LMICs, the VCPR currently focuses on children and adolescents with CP aged <18 years.<br><br>Adults are also likely under represented because in Vietnam, services and advocacy initiatives for people with CP have only recently been established and have historically focused on children. Diagnostic and support services for adults with CP are under development.<br><br>Furthermore, perceptions of disability have previously resulted in stigmatization of individuals with CP and their reluctance to access health and rehabilitation services and may account in part for the underrepresentation of adults.<br><br>I have clarified in the Limitations and Challenges as below:<br><br>"...the register may under-represent children with CP who are not connected with CPFAV or

	participating healthcare facilities and associations of people with disabilities, potentially impacting the completeness required for a population-based register. Establishing and maintaining a national register across multiple provinces will require ongoing coordination, training, and resources to support high-quality data collection and long-term sustainability. To improve ascertainment and identify the full range of severity of CP and to support the long-term sustainability of the register, the VCPR will incorporate community-based recruitment pathways alongside healthcare-based recruitment."(line 497-505)
**Comment 06 (Page 7; Line 2 - Introduction):** "... modeled on the ACPR and the GLM-CPR."<br><br>I don't believe the full term of GLM-CPR, Global Low and Middle Income Cerebral Palsy Register, has been mentioned before here, except in the Abstract section.	Thank you for this comment. I have added the full term, "Global Low- and Middle-Income Country Cerebral Palsy Register (GLM CPR)", at its first appearance in the Introduction section (Line 98).
**Comment 07 (Page 7; Line 29-34 - Introduction):**<br><br>"The VCPR is a partnership between Phenikaa University Hanoi, the Cerebral Palsy Family Association of Vietnam, the National Children's Hospital Hanoi and the University of Sydney. Collaborators include the Cerebral Palsy Alliance Australia, Central Queensland University, Hanoi Medical University and the Global LMIC CP Register."<br><br>I understand that the authors may wish to provide the full name of each party in this paragraph and I personally am not against this at all.<br><br>However, to improve readability, the authors may consider listing each full name followed by its abbreviation in parentheses, particularly for parties that are mentioned elsewhere in the manuscript, for instance:<br><br>"The Vietnam Cerebral Palsy Register (VCPR) is a partnership between Phenikaa University Hanoi, the Cerebral Palsy Family Association of Vietnam (CPFAV), the National Children's Hospital Hanoi (NCH Hanoi) and the	Thank you for this helpful suggestion. I have revised the manuscript accordingly by providing the full names followed by abbreviations at first mention, as suggested:<br><br>"The Vietnam Cerebral Palsy Register (VCPR) is a partnership between Phenikaa University Hanoi, the Cerebral Palsy Family Association of Vietnam (CPFAV), the National Children's Hospital Hanoi (NCH) and the University of Sydney. Collaborators include the Cerebral Palsy Alliance Australia, Central Queensland University, Hanoi Medical University and the Global Low and Middle Income Country Cerebral Palsy Register (GLM CPR)." (line 166 - 170)

University of Sydney. Collaborators include the Cerebral Palsy Alliance Australia, Central Queensland University, Hanoi Medical University and the Global Low and Middle Income Cerebral Palsy Register (GLM-CPR)."	
**Comment 08 (Page 7; Line 36-41 - Introduction):**<br><br>"Through our shared vision for optimal outcomes for people living with CP in Vietnam, our team of clinicians and researchers has established a strong relationship with CPFAV, including with Ms Dinh Thi Lan Anh, the CEO and founder of CPFAV, who was inspired by her journey with her daughter with CP to establish CPFAV. She will represent CPFAV in the partnership, including as a member of the VCPR Advisory Board."<br><br>I wonder whether this paragraph would fit better in the Acknowledgments section rather than here at the end of the Introduction.<br><br>Although the content itself seems appropriate, it appears somewhat disconnected from the surrounding context in the Introduction.<br><br>Therefore, the authors may consider keeping the paragraph unchanged but relocating it to the Acknowledgments section.	Thank you for this helpful suggestion. I agree that this paragraph fits better in the Acknowledgments section and have relocated it accordingly to improve the flow and readability of the Introduction.
**Comment 09 (Page 8; Line 40-42 - Methods: Register design and settings):**<br><br>"... children with CP aged <18 years..."<br><br>Is there a specific reason why only children younger than 18 years will be included in the future VCPR?<br><br>What are the major challenges or concerns associated with including individuals with CP who are 18 years of age or older?<br><br>Does the statement made later in the "Inclusion/exclusion criteria" section help	Thank you for this important comment.<br><br>The VCPR currently focuses on children aged <18 years because it is modelled on the GLM CPR, which collects data on children and adolescents with CP. This focus supports understanding of CP epidemiology, service needs, and outcomes during childhood, when interventions may have the greatest impact. It also facilitates data harmonisation and international comparisons.<br><br>In addition, the initial focus of the VCPR is to improve understanding of CP in childhood and inform early intervention, rehabilitation, healthcare planning, and policy development for children with CP in Vietnam. We acknowledge

explain the rationale behind this age restriction?<br><br>Even so, would including individuals with CP older than 18 years potentially raise any additional issues?	the importance of collecting data on adults with CP and will consider expansion of the register in future phases of development.
**Comment 10 (Page 9; Line 22-27 - Methods: Inclusion/exclusion criteria):**<br><br>"Any child… will be eligible for inclusion."<br><br>One major concern regarding the use of the aforementioned definitions or elements as inclusion/exclusion criteria, particularly for retrospectively acquired data, is that these criteria may rely on descriptions of past events in specific individuals.<br><br>To avoid potential contamination or misclassification in data inclusion, do the authors rely on other documentation, such as medical records from reliable sources or national health databases, to retrospectively confirm the diagnosis of each individual and maintain the accuracy of the registry?<br><br>If so, it would be helpful to elaborate on how this has been, or will be, implemented during the registry development process.	Thank you for this important comment.<br><br>To minimise the risk of misclassification, eligibility for inclusion in the VCPR will not rely solely on retrospective descriptions from families.<br><br>All participants registered through CPFAV are required to provide a medical record confirming a diagnosis of CP by a specialist clinician or hospital, which will be used to verify eligibility for inclusion in the VCPR.<br><br>For prospectively identified participants, data will be collected from children newly diagnosed with CP by a medical practitioner in participating healthcare facilities and/or children newly joining CPFAV with documented evidence of a CP diagnosis.<br><br>I have clarified this in the manuscript as below:<br><br>"For retrospectively identified participants, eligibility for inclusion in the VCPR will be verified using medical records documenting a diagnosis of CP made by a specialist clinician or hospital." (line 227 - 229)<br><br>Below information was also mentioned in the manuscript:<br><br>"Every CPFAV member with CP is required to provide a medical record that includes confirmation of a diagnosis of CP by a specialist clinician or hospital. With informed consent from families, electronic copies (photographs) of these records are obtained and securely stored at the CPFAV head office." (line 146 - 149)<br><br>"Subsequently, children newly diagnosed with CP by a medical practitioner in any healthcare facility and/or joining CPFAV will be enrolled in the VCPR." (line 241 - 243)

**Comment 11 (Page 10; Line 11-38 - Table 1):**<br><br>"Table 1. Children with CP registered in 63 CFPAV hubs in 34 provinces (N=4368; Updated 25/12/2025)"<br><br>1. The abbreviation is mis-spell as "CFPAV" instead of the correct "CPFAV."<br><br>2. The table is presented in ascending order according to the number of children available in each hub. What is the primary known reason for the difference in sample size (from 718 to 6) across hubs?<br><br>Is it related to the service capacity of each facility, the population size of the catchment area, or the socioeconomic status of the region?	Thank you for identifying this error. The abbreviation has been corrected from "CFPAV" to "CPFAV."<br><br>The variation in the number of children across CPFAV hubs is primarily related to differences in the timing of hub establishment and the population size of the areas they serve. The largest hub is located in Hanoi, where the CPFAV head office is based and where activities were first established. Importantly, these figures reflect CPFAV membership rather than CP prevalence.<br><br>I have clarified this in the manuscript as below:<br><br>“The number of children registered across CPFAV hubs varies because hubs were established at different times and serve populations of different sizes. The largest hub is located in Hanoi, where the CPFAV head office is based and where CPFAV activities were first established. The figures in table 1 reflect CPFAV membership rather than the prevalence of CP across provinces.” (line 256 - 260)
**Comment 12 (Page 11; Line 26 - #3):**<br><br>"The VCPR may contribute selected de-identified data to the Global LMIC CP Register..."<br><br>Which specific data elements will be selected for contribution to the GLM-CPR?	A minimum dataset, which is a subset of the data collected by the VCPR (i.e information on CP severity, aetiology, associated impairments, and functional outcomes), will be provided to the GLM CPR to enable comparisons and pooled data analyses where appropriate between LMICs.<br><br>I have clarified this in the manuscript as below:<br><br>“A minimum dataset, which is a subset of the data collected by the VCPR, will be provided to the GLM CPR. These core variables capture key information on CP severity, aetiology, associated impairments, and functional outcomes, enabling comparisons across LMICs and pooled data analyses where appropriate.21,37” (line 289 – 292)
**Comment 13 (Page 12-13 - Figure 1):**<br><br>Figure 1 is inserted here but is not mentioned anywhere in the main text.	Thank you for this helpful suggestion. We have now referred to Figure 1 in the Methods section and clarified its purpose as below:<br><br>“Figure 1 summarises the overall VCPR data collection process, including participant

Could the authors add a reference to Figure 1 in the relevant section and clarify what this figure is intended to illustrate?	identification, recruitment, data collection, clinical assessment, and data management procedures.” (line 429 - 431)
**Comment 14 (Page 14; Line 46 - Data collection):**<br><br>"For use by the VCPR, this form has been updated..."<br><br>It would be helpful if the authors could further specify what has been updated and explain the rationale for these updates.<br><br>This would help readers better understand the fundamental differences between Vietnam and other countries with established CP registries.	Thank you for this valuable suggestion. Through the co-design process, Vietnamese clinicians and parents identified several additional areas important to the local context, as well as variables that required adaptation. These modifications were made to better capture healthcare access, participation, and unmet needs among children with CP in Vietnam.<br><br>I have clarified this in the manuscript as below:<br><br>“For use in Vietnam, the VCPR Record Form was adapted through a co-design process involving Vietnamese clinicians and parents of children with CP (CPFAV members). Additional items included recent hospitalisations, common health conditions (e.g, sleep difficulties, constipation), and behavioural concerns.<br><br>Information on household composition, immunisation status and barriers to vaccination, educational participation and barriers to school attendance, and rehabilitation service utilisation and barriers to access is also collected. These variables are included in the GLM CPR and were adapted to align with the local context.<br><br>These modifications were considered important for understanding healthcare access, participation, and unmet needs among children with CP in Vietnam and for informing service planning, advocacy, and policy development.” (line 299-308)
**Comment 15 (Page 14; Line 17-34 - Data collection):**<br><br>"All caregivers of children... and other test results."<br><br>This is a critical yet challenging process in establishing the registry.<br><br>At this stage, do the authors have any estimates of the expected percentage of successful data collection at each stage for Part A and Part B?	Thank you for this important comment.<br><br>As this is a protocol paper, formal estimates of completion rates for Part A and Part B are not yet available, and no minimum data completion threshold has been pre-specified. The initial phase of the VCPR will provide important information on the feasibility of recruitment and data collection procedures.<br><br>To maximise data completeness, a dedicated hotline will be established to support CPFAV members who have questions, concerns, or

Alternatively, have the authors set a minimum target percentage of data completion that must be reached for the identified individuals at each stage?	difficulties during data collection and interview procedures. Researchers will also follow up on missing or unclear responses, and additional clinical information may be obtained through clinical assessment, consultation with healthcare professionals, or review of available medical records where consent is provided.<br><br>I have clarified this in the manuscript as below:<br><br>"Researchers will follow up on missing data or unclear responses to maximise data completeness. Additional clinical information may be obtained through clinical assessment, consultation with healthcare professionals, or review of available medical records with participant consent."(line 327 – 330)
**Comment 16 (Page 14; Line 44-47 - Data collection):**<br><br>"All data will be collected by qualified and trained doctors, physiotherapists and caregivers with a background in health, or qualified research assistants. Data collected will be reviewed weekly by senior investigators to ensure completeness and accuracy."<br><br>Could the authors further elaborate on why caregivers with a background in health will be included in the data collection process?<br><br>What types of professional backgrounds or specialties would be considered acceptable qualifications for a data collector?<br><br>In addition, are the "qualified and trained doctors, physiotherapists, caregivers with a background in health, or qualified research assistants" mentioned here the same as the data collectors described later on Page 16?	Thank you for these helpful comments. We apologise for the confusion caused by our description of the data collection process.<br><br>Clinical information (Part B of the VCPR Record Form) will only be collected by qualified and trained health professionals with expertise in CP, including paediatricians, rehabilitation physicians, physiotherapists, and general practitioners who have received specific training in CP assessment and data collection procedures. In contrast, Part A primarily comprises demographic, family, education, and service-use information and will be completed by caregivers, with support from trained research assistants where required.<br><br>The reference to "caregivers with a background in health" refers to CPFAV members who have health-related training and who may be recruited and trained as research assistants within their local hubs to support participant recruitment and data collection activities. They will not independently collect clinical assessment data.<br><br>I have clarified this as below:<br><br>"Part A of the VCPR Record Form will be completed by caregivers, with support from trained research assistants where required. Clinical information (Part B) will be collected only by qualified and trained health professionals, including paediatricians,

	rehabilitation physicians, physiotherapists, and general practitioners who have received training in CP assessment and VCPR data collection procedures. CPFAV members with a health-related background may be recruited and trained as research assistants to support participant recruitment and non-clinical data collection activities but will not conduct clinical assessments." (line 331 – 337)<br><br>Regarding data collectors described on Page 16 (curently page 18) THHK and THGN may participate in data collection activities. However, similar to the other individuals listed later in the manuscript, their primary roles are as senior investigators responsible for recruitment, data collection, and the overall conduct of the study in Vietnam.
**Comment 17 (Page 14; Line 48-50 - Data collection):**<br><br>"For children enrolled in the VCPR before the age of five years..."<br><br>What is the purpose of re-contacting families when the child reaches five years of age?<br><br>In addition to this five-year follow-up, do the authors expect to re-contact the family at any later stage of the individual's life?	Thank you for this important question.<br><br>Children enrolled before five years of age will be re-contacted at age five years to confirm the CP diagnosis and update clinical information because diagnosis, classification, severity, and associated impairments become clearer with age. This approach is consistent with established CP registers, including the Australian Cerebral Palsy Registers.<br><br>The current VCPR protocol includes a single planned follow-up when the child reaches five years of age. No additional routine follow-up is currently planned.<br>I have clarified this in the manuscript as below:<br><br>"For children enrolled in the VCPR before the age of five years, families will be re-contacted when the child reaches five years of age to confirm the CP diagnosis and review previously collected clinical information. This follow-up recognises that the diagnosis, classification, severity, and associated impairments in CP may become clearer with age and helps minimise potential misclassification."(line 338 – 343)
**Comment 18 (Page 15; Line 43-44 - Variables: Associated features):**	Thank you for this important suggestion. We agree that skeletal deformities in children with GMFCS III-V make standard growth assessments challenging. We have clarified in the manuscript

"... growth and nutritional status (Z-scores and percentiles for current weight for age, height for age,..."<br><br>The authors do not, and certainly should not, restrict inclusion in the VCPR based on GMFCS level.<br><br>However, it is well recognized that, in children with CP classified as GMFCS Levels III-V, accurate assessment of growth based on standing body height or arm span can be challenging because of moderate to severe skeletal deformities involving the lower limbs and spine.<br><br>Would the authors consider including additional anthropometric measurements that may better reflect actual growth status in this population?	that when standard measurements are unfeasible, specialized alternative methods are used to reflect actual growth status.<br><br>I have clarified this in the manuscript as below:<br><br>"Associated features: growth and nutritional status, including Z-scores and percentiles for current weight for age, height for age, weight for height. For children with severe contractures or scoliosis (GMFCS III-V) where standing height is difficult to obtain, we acknowledge that segmental measures (e.g., knee height) or mid-upper arm circumference are preferred alternatives to better reflect actual growth status.38,39 Head circumference, growth delay, gastrointestinal and nutritional problems (GERD, constipation, dental caries), and musculoskeletal complications (e.g. contractures, scoliosis, hip dislocation) are also recorded."(line 373 - 380)
**Comment 19 (Page 15; Line 43-44 - Variables: vaccination status):**<br><br>"... vaccination status (fully vaccinated or not according to the EPI for Vietnam), receipt of specific vaccines, reason for missed vaccines, presence of BCG scar."<br><br>Each country may have its own vaccination regulations and schedules, depending on geographic location, public health policy, and historical context.<br>It may be helpful for the authors to provide appendix information on the current vaccination regulations in Vietnam, including vaccine types and recommended timetables, to help future readers better interpret the results.	Thank you for this helpful suggestion. We have added the current EPI schedule, including vaccine types and recommended ages for administration, as Supplementary file.<br><br>I have clarified this in the manuscript as below:<br><br>"Details of the current EPI for Vietnam,40 including vaccine types and recommended vaccination schedules, are provided in Supplementary file 2." (line 385 – 387)
**Comment 20 (Page 16; Line 10-17 - Data collectors):**<br><br>"Training of data collectors"<br><br>I assume that the recruited data collectors will be responsible for collecting data from NCH.	Thank you for this important question.<br><br>The data collectors will not be limited to staff of the NCH. As the VCPR is a national register, data collection will be undertaken by qualified and trained doctors and physiotherapists from NCH, CPFAV, and other participating healthcare facilities across Vietnam.

Will they also be involved in data collection on the CPFAV side?<br><br>Based on the current plan, how many data collectors are expected to be required to manage the workload of this project?	The exact number of data collectors will depend on participation rates. During the establishment phase, a trained clinical data collector may support multiple CPFAV hubs. As the VCPR expands, our goal is for each CPFAV hub (total 63 hubs across Vietnam) to have access to at least one trained clinical data collector within the first year of operation.
**Comment 21 (Page 19; Line 10-17 - Data entry / Page 8; Line 41 / Page 19; Line 46-47):**<br><br>"... will be entered into the CPRV database..."<br><br>This is the only instance in which the abbreviation "CPRV" is used throughout the protocol. Could this be a typographical error, and should it instead refer to "VCPR"?<br><br>Page 8; Line 41: "... CPVR Advisory Board."<br><br>Page 19; Line 46-47: "We will regularly report the results of CPVR data analysis..."<br><br>Same as above, should these two abbreviations be changed to "VCPR" instead?	Thank you for identifying these typographical errors. We confirm that "CPRV" and "CPVR" should be "VCPR". These errors have now been corrected throughout the manuscript.
**Comment 22 (Page 17; Line 12 - Expert Advisory and Investigative Team):**<br><br>"... The Vietnam Cerebral Palsy Register (VCPR) will be led by..."<br><br>The full form of VCPR may not be necessary here.<br><br>Using the abbreviation alone should be sufficient: "The VCPR will be led by..."	Thank you for this suggestion. We agree and have revised the text accordingly.
**Comment 23 (Page 4; Line 20-22 - Strength and limitations of this study):**<br><br>"Potential selection bias, with under-representation of children not accessing hospitals or CPFAV networks."<br><br>Although this potential limitation has already been noted as the only one in this section on Page 4, it would be helpful if the authors could include a separate "Limitations and Challenges" section near the end of the protocol, possibly after Dissemination on	Thank you for this helpful suggestion. We agree that a dedicated section on limitations and challenges will assist readers in interpreting the findings and understanding the context of the VCPR. We have therefore added a "Limitations and Challenges" section to the manuscript.<br><br>"As with any registry, the VCPR has several limitations and challenges that should be acknowledged. As participation in the VCPR is voluntary, the register may under-represent children with CP who are not connected with CPFAV or participating healthcare facilities and

Page 17 and before Authors' Contributions on Page 18.<br><br>This section could further discuss known limitations related to the current study design, the restriction from the Vietnamese clinical and research environment, and any anticipated challenges in establishing the VCPR.	associations of people with disabilities, potentially impacting the completeness required for a population-based register. Establishing and maintaining a national register across multiple provinces will require ongoing coordination, training, and resources to support high-quality data collection and long-term sustainability. To improve ascertainment and identify the full range of severity of CP and to support the long-term sustainability of the register, the VCPR will incorporate community-based recruitment pathways alongside healthcare-based recruitment." (line 496 – 505)

VERSION 2 - REVIEW

**Reviewer**	**1**
**Name**	**Chen, Brian Po-Jung**
**Affiliation**	**Chang Gung Memorial Hospital Linkou**
**Date**	**03-Jul-2026**
**COI**	

I would like to thank the authors for their careful and thoughtful revision. The authors have clearly addressed my concerns and responded thoroughly to all of my comments. I have very much enjoyed this constructive exchange and sincerely appreciate the effort that has gone into improving the manuscript. The study protocol is carefully designed, and the manuscript is clearly and elegantly written. Based on the revised version (R1), I have no further questions or concerns.

Coordinating and establishing a registry for this population in a low- or middle-income country is understandably challenging. I sincerely hope that the authors will be able to successfully carry out this study protocol and establish the Vietnam Cerebral Palsy Register. This valuable experience will help pave the way for other regions or countries seeking to develop their own cerebral palsy registries in the future. I wish the authors every success with this important work.
